# A Systematic Review of Tissue and Single Cell Transcriptome/Proteome Studies of the Brain in Multiple Sclerosis

**DOI:** 10.3389/fimmu.2022.761225

**Published:** 2022-03-02

**Authors:** Maria L. Elkjaer, Richard Röttger, Jan Baumbach, Zsolt Illes

**Affiliations:** ^1^ Department of Neurology, Odense University Hospital, Odense, Denmark; ^2^ Institute of Clinical Research, University of Southern Denmark, Odense, Denmark; ^3^ Institute of Molecular Medicine, University of Southern Denmark, Odense, Denmark; ^4^ Department of Mathematics and Computer Science, University of Southern Denmark, Odense, Denmark; ^5^ Chair of Computational Systems Biology, University of Hamburg, Hamburg, Germany

**Keywords:** multiple sclerosis, systems biology, transcriptome, proteome, single cell, brain lesions, NAWM, NAGM

## Abstract

Multiple sclerosis (MS) is an inflammatory demyelinating and degenerative disease of the central nervous system (CNS). Although inflammatory responses are efficiently treated, therapies for progression are scarce and suboptimal, and biomarkers to predict the disease course are insufficient. Cure or preventive measures for MS require knowledge of core pathological events at the site of the tissue damage. Novelties in systems biology have emerged and paved the way for a more fine-grained understanding of key pathological pathways within the CNS, but they have also raised questions still without answers. Here, we systemically review the power of tissue and single-cell/nucleus CNS omics and discuss major gaps of integration into the clinical practice. Systemic search identified 49 transcriptome and 11 proteome studies of the CNS from 1997 till October 2021. Pioneering molecular discoveries indicate that MS affects the whole brain and all resident cell types. Despite inconsistency of results, studies imply increase in transcripts/proteins of semaphorins, heat shock proteins, myelin proteins, apolipoproteins and HLAs. Different lesions are characterized by distinct astrocytic and microglial polarization, altered oligodendrogenesis, and changes in specific neuronal subtypes. In all white matter lesion types, *CXCL12, SCD, CD163* are highly expressed, and STAT6- and TGFβ-signaling are increased. In the grey matter lesions, TNF-signaling seems to drive cell death, and especially *CUX2*-expressing neurons may be susceptible to neurodegeneration. The vast heterogeneity at both cellular and lesional levels may underlie the clinical heterogeneity of MS, and it may be more complex than the current disease phenotyping in the clinical practice. Systems biology has not solved the mystery of MS, but it has discovered multiple molecules and networks potentially contributing to the pathogenesis. However, these results are mostly descriptive; focused functional studies of the molecular changes may open up for a better interpretation. Guidelines for acceptable quality or awareness of results from low quality data, and standardized computational and biological pipelines may help to overcome limited tissue availability and the “snap shot” problem of omics. These may help in identifying core pathological events and point in directions for focus in clinical prevention.

## 1 Introduction

Multiple sclerosis (MS) is a common cause of neurological disability among young adults that evolves in clinically different stages termed radiologically isolated syndrome (RIS), clinically isolated syndrome (CIS), relapsing-remitting MS (RRMS), secondary progressive phase (SPMS), and primary progressive MS (PPMS). However, this classification may not directly reflect the pathological mechanisms similarly to another classification that only considers clinical/radiological activity and disability progression (1).

MS has a heterogeneous, multifactorial origin that involves interactions between the immune and nervous system impacted by the genetic background (2) and by the environment (3, 4). The main pathological features are accumulation of lesions in the grey and white matter (GM, WM). These are characterized by different degrees of inflammation, demyelination, neuronal and axonal degeneration, oligodendrocyte loss, gliosis/glia activity, and remyelination. Additional features are diffuse inflammation in the normal-appearing (NA) tissues, meningeal infiltrates, and global CNS atrophy (5). Especially in early relapsing MS, influx of systemic immune cells into the CNS induces inflammatory demyelinating lesions (6, 7). As the disease progresses, the number of chronic active lesions increases, and they inversely correlate with the number of remyelinating/repairing lesions (8–10). Lesions in cortical and deep GM areas and neuronal loss become prominent in the progressive phase (11). At this stage, inflammation becomes more compartmentalized and is governed primarily by microglia, astrocytes, and tissue-resident lymphocytes (12, 13).

Approved MS treatments impact systemic adaptive immune responses and work effectively in the early phase (14). However, their passage through the blood-brain barrier is limited, and most of them do not affect innate immune responses in the CNS. Their effect on compartmentalized immune responses is largely unknown. Such limitations are also reflected by their poor impact in the progressive phase. Neuro- and oligodendrocyte-protective treatments that inhibit or reverse degenerative processes are basically missing. To develop efficient treatments for the progressive phase, understanding the molecular mechanisms of pathological events within the CNS is essential. This has shifted focus of MS research to CNS-specific events. Recent advances in omics will hopefully integrate several levels of spatiotemporal data, and may help to understand, how multiple factors can converge into phenotypically similar disease states. Such knowledge may also fuel novel treatments (15, 16). To accomplish such goals, several challenges have to be overcome, e.g. experimental and computational pipelines have to be standardized, and large amount of descriptive biological data should be functionally interpreted. Here, we systemically review the transcriptome and proteome studies in the MS brain and discuss gaps and obstacles.

## 2 Methods

### 2.1 Search Strategies

A systematic electronic search was conducted in PubMed with the following search terms from as far back as possible (earliest identified study was from 1997) to October 2021: category one “multiple sclerosis”; category two “brain”, “lesions”, “white matter”, “grey matter”; category three “omics”, “profiling”, “transcriptome”, “array”, “next generation sequencing” “proteome”; category four “human” and NOT “review”. The search was also complemented by reference lists of articles identified by this search strategy.

### 2.2 Selection Criteria

Studies were included, if they fulfilled the following criteria: (i) the study was performed on human brain tissue from patients with MS; (ii) the study used next-generation sequencing, mass spectrometry or arrays on the human brain tissue; (iii) article written in English.

Studies were excluded if the study design was not clearly stated.

## 3 Results

**Figure 1:**
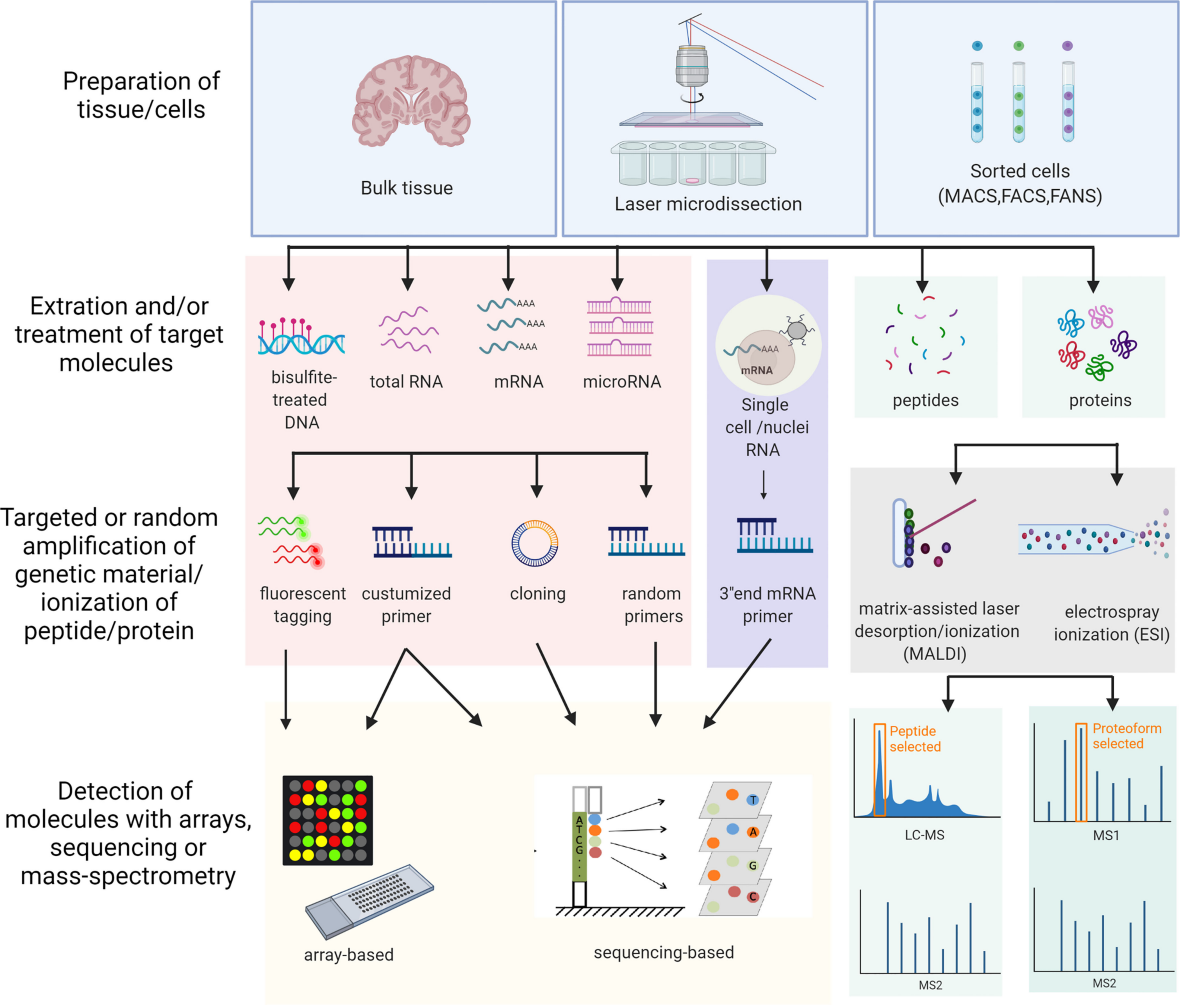
Schematic overview of the different omics approaches used in the study. The overview includes all the different methods used in the studies included in this review. MACS, Magnetic-activated cell sorting; FACS, Fluorescence-activated cell sorting; FANS, Fluorescence-activated nucleus sorting; LC-MS, Liquid chromatography–mass spectrometry. Created with BioRender.com.

**Figure 2:**
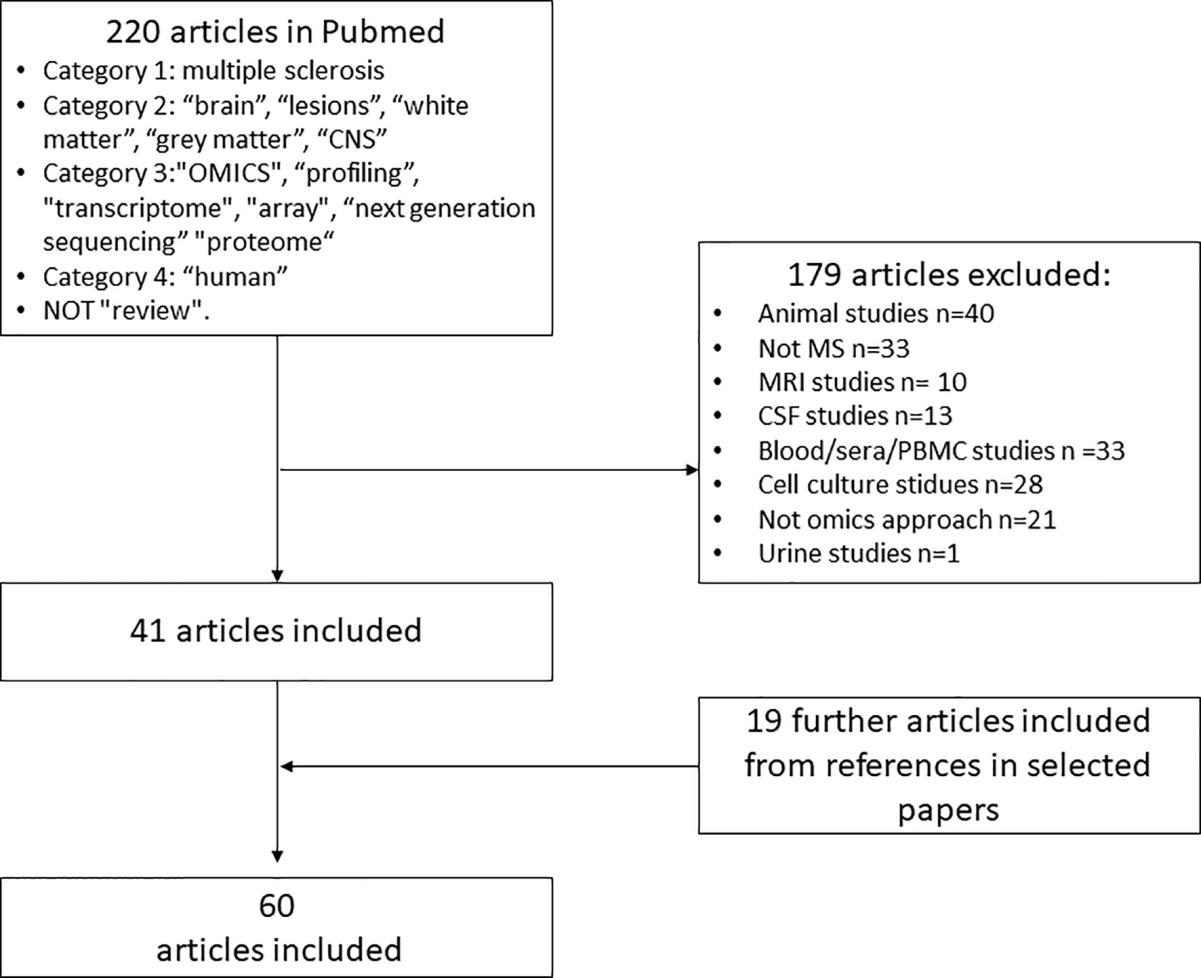
Flowchart for identification and inclusion of relevant studies for systematic review. PMD, postmortem-delay; RIN, RNA integrity number.

Omics studies on MS brain tissue are few. An overview of the different methods is illustrated in **Figure 1**. Advantages and disadvantages of different omics techniques are listed in **Table 1**. A flowchart summarizing the identification of relevant studies according to PRISMA is presented in **Figure 2**.

**Table 1:** An overview of the advantages and disadvantages of the omics techniques.

Omics	Target	Definition	Technology	Application	Temporal variance	Disadvantages	Advantages
Genomics	DNA	Assessment of variability in the DNA sequences of the genome	Whole genome sequencing	Genome-wide mutational analysis	None	Limited information about the MS state and prognosis	SNP variability is stable during life
			Exome sequencing (1.5% of the genome)	Exome-wide mutational analysis		Limited information about the MS state and prognosis, only information within the exons	
Epigenomics	Molecular changes on the DNA	Assessment of variability of factors that regulate the genome without changing the DNA sequence	WGBS (whole genome bisulfite-treated DNA sequencing)	Methylome-wide pattern and alterations	Moderate	Complex data analysis, lack of functional knowledge on methylation at other sites	Whole methylome state on single base pair level
			RRBS (bisulfite-treated CpG enriched region sequencing (3% of the genome))	Methylome pattern of CpG enriched regions based on restriction enzymes		Missing areas, difficulties in comparing between samples due to unpredictable cleavage and enrichment, no information at other bases (A,T, C)	Focused methylation status at CpG regions
			TBS (bisulfite-treated hybridized target DNA region sequencing)	Targeted methylation analysis of selected candidate genes		Need prior knowledge on candidate areas	Parallel investigation of many candidate genes
			Microarray (hybridization of ~850,000 probes at methylation sites)	Interrogation of pre-selected methylation sites across the genome		Limited to the probes available, no information at other bases (A,T, C), high background noise, not fully compatible across platforms	Cost efficient, methylome of 95% of CpG islands, high coverage of enhancer regions
			ATAC-seq (Tn5 transposase treated DNA sequencing)	Identification of accessible chromatin regions in genome- wide, including transcription factors, histone modifications.		Time-consuming, poor repeatability, signal-to-noise ratio is low	Unbiased identification of a real time profile of all active regulatory sequences in the genome using a small amount of cells
			ChIP-seq (chromatin immunoprecipitated DNA sequencing)	Analyze protein interactions with DNA by genome-wide mapping of epigenetic marks, transcription factors, or other DNA-binding proteins		Require good antibody for target protein, high amount and high quality of tissue	Map global binding sites precisely for any protein of interest, analyze the interaction pattern of any protein with DNA, or the pattern of any epigenetic chromatin modifications
			Sc/snATAC-seq (Tn5 transposase treated DNA sequencing within intact single nuclei)	Identification of accessible chromatin regions within single cells		Require high quality tissue, unclear if it is a limited subset of open chromatin sites in single cells	As ATAC-seq, but provides examination of cell-to-cell variability in chromatin organization,
Transcriptomics	Activated genes/RNA	Assessment of variation on composition and abundance of the transcriptome	Microarray (cDNA hybridization of targets of interest to probes)	Differential gene expression analysis of protein-coding-genes (~18,700) or designed probes of interest	High	Limited dynamic range (probe-dependent), problems with competitive hybridization, high background, low sensitivity, not fully compatible across platforms	Well-defined protocols and analysis pipelines
			Next generation RNA-seq (cDNA sequencing of RNA with rRNA removal or mRNA enriched)	Genome-wide differential gene expression analysis of total RNA or mRNA		PCR amplified biases, lack of standardization between sequencing platforms (effect dynamic range and reproducibility), do not capture the whole transcriptome (small drop-outs)	Unbiased insight into all transcripts (novel and non-coding), accurately measuring expression level changes, ability to detect expression changes in non-coding genes
			EST (expressed sequencing tags of randomly selected clones sequenced from cDNA libraries (total RNA or poly (A) RNA))	Differential gene expression analysis of the partial mRNA pool of the sample		Only partial profiles of the gene expression, a large numbers of housekeeping genes, neglect rare transcripts	Suitable for gene discovery, rapid and easy protocols
			Amplicon (targeted sequencing based on probes designed for targets of interest)	Differential gene expression analysis of targets of interest		Prior knowledge of target RNAs	Multiplexing of hundreds to thousands of amplicons per reaction, less sequencing with high coverage
			Sc/snRNA-seq (poly(A) tagging, 5′-end, 3’-end or total RNA-sequencing within intact single nuclei or cell)	Gene expression profiles of individual cells		More time-consuming, require high quality tissue, identifies fewer transcripts than bulk RNA-seq (high drop-out), imperfect coverage can lead to a biased quantification, complex analyses	Transcriptomic profiling of heterogeneous tissue, or dynamic processes in single and within cell groups, sensitive, interrogate nuances of cell signaling pathways
			Spatial transcriptomics (sequencing of released tissue mRNA captured on spotted histology slides to combine gene activity with spatial resolution)	Spatially-resolved transcriptomics		Intact good quality tissue block, not single cell level (each spot represent 10-100 cells), complex analyses, time-consuming, good microscope	Map out gene expression in spatial context, capture how gene expression data might reflect the spatial relationships among multiple cells
Proteomics	Proteins	Assessment of variation on composition and abundance of the proteome	Mass spectrometry (identify (u)known peptides/proteins via separation of gaseous ions according to their differing in mass and charge)	Identification and quantification of proteins in a sample	High	Time-consuming complex data analysis, protein detection is affected by high abundance proteins and peptide ionization	Incredibly sensitive (parts per million), excellent for identifying unknown components or confirming their presence and abundance
			Array (binding of targets of interest to peptides (up to tens of thousands in several copies))	Identification and quantification of proteins of interest in a sample		Limited to prior knowledge (not discovery)	Profiling multiple proteins without disturbance of high abundance proteins, high number of arrays available for a wide range of applications.
			Sc mass cytometry (simultaneous measurement of more than 40 proteins at single-cell resolution)	Multiplexed and quantitative measurements of proteins and their modifications on single cells		Low dimension, prior knowledge of targets, limited target number (40), significant variation in signal intensity over time and across machines	Highly multiplexed and quantitative measurements of proteins and modifications, good pipelines for analysis

### 3.1 Transcriptional (mRNA, ncRNA, MicroRNA) Approaches to Examine Pathological Mechanisms in the MS Brain Tissue

In the late 1990s, the first large-scale gene expression profiles were performed on different WM lesions from both autopsies and biopsies using microarrays (**Table 2**). They revealed alterations in cell metabolism, shifts in cytokines and cell adhesion molecules (17, 18), new inflammatory (19, 20, 44) and oxidative damage markers (23). In the 2010s, the number of samples increased, and microdissected tissues were also analyzed in designed systems biology studies; these included vessels near lesions (27), chronic active rim areas (33), or specific cell types like astrocytes (32). Single-cell/nucleus technologies, such as single-cell/nucleus RNA sequencing (sc/nRNA-seq, spatial-seq) that promote identification of novel cell types and cell state transitions have been published since 2019 (56–58).

**Table 2:** An overview of the studies (n=49) that examined the transcriptome profile in human MS brain tissue.

**Microarray of tissue (laser captured or macrodissected) and isolated cells: mRNA**						
**Authors**	**Patients**	**Quality** **(PMD, RIN)**	**Methodology**		**Key findings**	
Whitney et al. (17)	- 2 lesions from Becker et al. (43) - 1 NAWM from same patient	PMD: 8h	Tissue mRNA array		- 20 DEGs in lesion vs. NAWM related to cell metabolism, cytokines and cell adhesion molecule.	
Baranzini et al. (18)	- 8 MS samples with active demyelination - 8 controls (non-MS)	–	Tissue mRNA array		- 31 DEGs in MS. - *CD4* was the most overexpressed gene. - Predominant expression pattern of Th1 cytokines mainly represented by *MIP-1a, RANTES, caspase-1, IL-1B, IL-18 IL-5, IL-6.*	
Whitney et al. (19)	- 2 lesions from PPMS [from Becker et al. (43)] - 1 RRMS with chronic silent lesion - NAWM from the two patients	–	Tissue mRNA array		- *Arachidonate 5-LO* overexpressed in both microarray and EAE disease states but not NAWM or normal mouse brain.	
Lock et al. (20)	- 1 active, 3 chronic active, 3 chronic inactive from 4 progressive MS patients - 2 control subjects	PMD: 1.5-8h	Tissue mRNA array		- *MAPK2* and *GM-CSF* were higher expressed in acute than chronic active lesion. - *FcRy* was higher expressed in chronic than acute lesion.	
Graumann et al. (21)	- 12 NAWM in 10 MS - 8 WM in 7 control subjects	PMD: 5-22h	Tissue mRNA array		- DEGs in NAWM were involved in energy metabolism, neuroprotection, oxidative stress and ischemic preconditioning, axonal transport and synaptic transmission: *HIF1a, CREB, PI3K/Aktm VEGF, hexokinase 1, Cl-transporter, adenosine A1 receptor, GABA-A/B R, 14-3-* *3, STAT6+MCSF, IL-1, TNFa and GSH, ROS/RNS NF-L NF-M, synaptophysin, SCG10.*	
Mycko et al. (22)	- 2 chronic active (marginal and centre) and 2 silent (marginal and centre) lesions from 4 SPMS	PMD: <8h	Tissue mRNA array		- Pathological events differ in the centre and at the edge of the chronic lesions. - 9 DEGs in in the marginal zone of chronic active lesions were highlighted: *CD4, IFNg, MAPKK1, Caspase 9, Cbl-b, EDDR1, HSP90, FLT3 ligand, adenosine A1 receptor.*	
Tajouri et al. (23)	- 2 acute and 3 chronic active lesions from 5 SPMS - 4 control areas from non-MS	PMD: 4-24h	Tissue mRNA array		- Upregulation of immune-related DEGs: *MAL, VIL2, CXCL10, CXCR3* in MS. - Detection of genes related to oxidative damage protection: *TF, SOD1, GPX1, GSTP1.*	
Lindberg et al. (24)	- 5 active lesions and 5 NAWM lesions from 6 SPMS - 12 WM from 12 control subjects	PMD: 3:45-9:20h	Tissue mRNA array		- Lesions and NAWM shared downregulated DEGs of anti-inflammatory property*: EGFR*, *TGFB3, cre-bp-1.* - Lesions differed from NAWM by higher *Ig* level and *IL-6R.* - Lesions had DEGs related to neuroglial development: *NF-L/M, STMN2, a/b-tubulin, dynamin, CAP2.*	
Mycko et al. (25)	Same data as Mycko et al. (22)	PMD: <8h	Tissue mRNA array		- The centre of chronic active and inactive lesions had fewer genes differentially expressed and less infiltration. - *TNF* and *IL-6* were underrepresented in chronic inactive, but upregulation of *bcl-xm GFR2, hsp90A hsp60.*	
Zeis et al. (61)	- 11 NAWM from 11 MS - 8 controls	PMD: 6-26h	Tissue mRNA array		Upregulation of both pro-inflammatory response: *STAT4, IL-1B, MCP-1, ICAM-1, RANTES, HLA-DR*; and anti-inflammatory response: *IL-10, TGFB2, STAT6, IL4R, IL13R.*	
Zeis et al. (26)	- 4 biopsy from both lesion and non-demyelination in MS patient - 8 NAWM autopsy MS patients - 2 biopsy controls	–	Tissue mRNA array		- Active astrocytes (*GFAP, AQP4, HLA-DRA*) and active oligodendrocytes (*PLP, MAG,STAT6, nNOS, HO-1*) are strongly up-regulated in non-demyelinated WM during a very early acute phase of MS.	
Cunnea et al. (27)	- Chronic active, chronic inactive and NAWM from 4 PPMS and 8 SPMS - WM from 5 controls	PMD: 8-33h	Microarray of microdissected vessels		- 113 genes involved in all aspects of endothelial cell biology, and 50% of those were DEGs from chronic active or inactive compared to NAWM or control. - Upregulated genes in chronic active and inactive were among others *VEGFA, MMP1*, *MMP14* and *ICAMs.*	
Fischer et al. (28)	3 microdissected active lesions of patients with fulminant acute MS	–	Tissue mRNA array		Array detected genes of mitochondrial injury together with gene expression of various nicotinamide adenine dinucleotide phosphate oxidase subunits. The data suggest inflammation-associated oxidative burst in activated microglia and macrophages.	
Mycko et al. (29)	5 CA lesions (marginal and centre) compared with NAWM from 5 SPMS	PMD: <8h RIN:6-7.5	Tissue mRNA array		- 45 heat-shock protein (HSP) genes of all 8 major families were present, and the pattern of HSP differed between centre and margin of the chronic active lesions.	
Mohan et al. (30)	- 6 demyelinated inactive lesion from 4 MS - 4 remyelinated lesions from 3 MS - 4 demyelinated active lesions from 3 MS - 6 WM from 4 controls	–	Tissue mRNA array		- *FGF1* was the most abundant gene in remyelinating lesions compared to demyelinating and WM control tissue.	
Licht-Mayer et al. (31)	WM study: - 4 acute MS cases each with NAWM, initial demyelinating lesions, late active lesions - 4 control casesGM study: - 3 SPMS each with cortical lesions - 3 control cases	–	Tissue mRNA array		- Nrf2 is upregulated in active MS lesions, especially in oligodendrocytes, while few number of Nrf2-postive neurons were detected. - A number of Nrf2-responsive genes involved in protection against oxidative stress were upregulated in initial demyelinating lesions. - Expression pattern of Nrf2-induced genes differed between WM and GM.	
Waller et al. (32)	- 5 samples with astrocytes in NAWM from MS - 5 samples with astrocytes in WM from controls	PMD:5-33h RIN:>3	mRNA array of GFAP positive cells		Genes upregulated in NAWM astrocytes were related to scavenge transition metal ions and free radicals (*MT1,MT2*), transport and storage of iron (*FTL, TF*) and immune related ischaemic preconditioning (*TGF-B3, MAPKAPK2, MAPK4*), while gene encoding COX2 enzyme (*PTGS2*) was downregulated.	
Hendrickx et al. (33)	- rim and perilesional-NAWM of 7 chronic active and 8 inactive lesions from 12 RRMS, 1 PPMS, and 2 with unknown MS disease course - 10 WM from 10 control subjects	PMD: 8:23±2.51-9:03±0.45h RIN: 5.79±0.62-7.42±0.67	Tissue mRNA array		- Upregulation of DEGs in rim of lesions involved in immune function, lipid binding, lipid uptake, and neuroprotective functions - Identified a set of genes that are related to lesion activity and expansion*: CHIT1, GPNMB, CCL18, OLR1, CD68, MSR1, CXCL16, CXCR4, NPY, KANK4, NCAN, TKTL1, ANO4*.	
Zeis et al. (34)	- 9 active lesions, 9 NAWM, 7 remyelinating lesions and 5 inactive lesions from 7 PMS patients	PMD: 9-27h RIN:>7	Tissue mRNA array		- Increased expression of STAT6-singaling gens in active, remyelinating and inactive lesions - Expression of genes involved in oligodendrogliogenesis were qualitative and quantitative differently expressed in the different WM lesions	
Melief et al (35)	- NAWM from 18 MS - WM from 9 controls	PMD: 4:15-13:20h RIN: 7.4-7.8	Tissue mRNA array		In MS patients with mild MS and high HPA-axis, the NAWM expression profile reflected genes involved in regulation of inflammation, myelination, anti-oxidant mechanisms and neuroprotection.	
Magliozzi et al. (36)	- 20 MS motor cortex with and without substantial meningeal inflammation - 10 controls	PMD: 3-44h RIN: >7	Tissue mRNA array		A changing balance of TNF signalling in the cortex depending on the degree of inflammation.	
Enz et al. (37)	64 NAGM samples of 25 MS patients and 42 control GM samples of 14 controls	PMD: 3-28h RIN: >6	Tissue mRNA array		*HLA-DRB1* is significantly higher expressed in MS NAGM and the protein expression is increased in HLADRB1*. 15:01-positive cases in grey matter on microglia based on immunofluorescence colocalization.	
Jäckle et al. (38)	- 8 chronic active, 8 NAWM and 1 lesion rim af a chonic inactive lesion	PMD: 9-34h RIN: >3	Tissue mRNA array		- Accumulation of M1 microglia phenotype at lesion rim. - Upregulation at *ALOX15B, MME* and *TNFRSF25* in the lesion rim.	
**Microarray of tissue (laser captured or macrodissected): microRNA and methylome**						
**Authors**	**Patients**	**Quality** **(PMD, RIN)**	**Methodology**		**Key findings**	
Junker et al. (39)	- 16 active and 5 inactive white matter multiple sclerosis brain lesions - 9 control white matter specimens.		Tissue microRNA array		- miRNA signatures of active and inactive brain lesions of patients with MS. - microRNA-34a, microRNA-155 and microRNA-326 were upregulated in active MS lesions and related to the CD47 in microglia/macrophages.
Chomyk et al. (40)	9 myelinated and 7 demyelinated regions of hippocampus from 15 MS patients	PMD: 4-12h	Tissue methylation array		Genes involved in synaptic plasticity and neuronal survival were altered by methylation changes following demyelination in MS hippocampus. Here among hypomethylation of 6 genes (*AKNA, EBPL, FLJ42709, HERC6, OR52M1, SFRP1*) in demyelinated regions.	
Tripathi et al. (41)	5 myelinated and 5 demyelinated WM lesions 6 SPMS patients	PMD: 9-37h	Tissue microRNA array		- Discovery of 11 pathogen-related and 12 protection-related miRNAs previously identified in sera and correlating with WM MRI abnormalities. - 7 of the 12 microRNAs related to protection were decreased in the MS lesions.	
Kular et al. (42)	- Neuronal nuclei isolated from 14 MS patients (incl. NAWM, active, chronic active, chronic lesions) and 12 controls	PMD: 11± 11.4-23±3.7h	Tissue methylation array		- DNA methylation alterations in WM-neurons from MS patients compared to control. - Potential impaired CREB-mediated neuro-axonal integrity due to hypo-5mC and hyper- 5hmC in MS neurons.	
Fritsche et al. (64)	- 7 subpial lesions, 7 leucocortical lesions, 7 chronically inactive WM lesions and NAWM from 18 MS brains - Subpial and leucocortical areas of normal GM and normal WM from 12 age-matched controls		Tissue microRNA array		- 5 of 7 significantly upregulated miRNAs in grey matter lesions (miR-330-3p, miR-4286, miR-4488, let-7e-5p, miR-432-5p) shared the common target synaptotagmin7 (Syt7).	
Tripathi et al. (41)	miRNA study: 5 NAGM and 5 MS demyelinating cortical lesions mRNA study: 8 NAGM from 6 MS brains and 8 cortical lesions from 8 MS brains	PMD: 3-9h	Tissue microRNA array		- 10 significant up- and 17 significant downregulated microRNAs in demyelinated GM vs. NAGM. - Predicted target mRNAs belonged to TGF-β signalling and FOXO signalling. - mir149, mir20a, mir29c and mir24 were key regulators based on PPI network analysis.	
**Next generation sequencing (NGS) of tissue (laser captured or macrodissected) and isolated cells: mRNA and total RNA**						
**Authors**	**Patients**	**Quality** **(PMD, RIN)**	**Methodology**		**Key findings**	
Becker et al. (43)	- 3 lesions from 1 PPMS - 2 areas from healthy adult brain	PMD: 8h	Expressed sequencing tag (EST)	- 56 DEGs related to immune activation in PPMS. - Discovery of *MIP-1a* and *RANTES*.		
Chabas et al. (44)	- 2 acute and 1 chronic lesion from 3 MS patients - 1 control subject		EST	- 50 DEGs in MS as *GFAP, MBP, HSP70, CRYAB* and *OPN* (osteopontin). - Degree of *OPN* expression correlated with severity of EAE disease.		
Schmitt et al. (45)	- 7 WM lesions from 6 MS - 7 WM areas from 7 controls	PMD: 4:50-12h	Next generation amplicon sequencing		- No significantly different transcription patterns, when comparing HERV-W transcription in brain lesions from MS to healthy.	
Huynh et al. (46)	- 28 NAWM from MS - 19 WM from controls	PMD: ≥31h RIN: ≥7	Tissue NGS (mRNA) and methylation array		- Downregulated and hypermethylated genes in NAWM were related to oligodendrocyte and neuronal function (*BCL2L2, HAGHL, NDRG1*). - Upregulated and hypomethylated genes in NAWM were encoding for cysteine proteases (*CTSZ, LGMN*).	
Kriesel et al. (47)	Frozen brain tissue from: - 14 demyelinating brains: PPMS (n=11), SPMS (n=1), NMO (n=2) - 14 controls - 7 OND: herpes encephalitis (n=3), unknown encephalitis (n=2), subacute sclerosing pan encephalitis (n=2)	PMD: 4-24h	Tissue NGS (total RNA)		- Overexpression of HERV in demyelinating and OND brain samples compared to normal brain. Specific HERV and KRAB sequences were overexpressed in the demyelinating group.	
Elkjaer et al. (48)	- 21 NAWM, 16 active, 17 chronic active, 14 inactive, 5 remyelinating lesion from 10 progressive MS patients - 25 WM of non-neurological disease subjects	PMD: 8-30h RIN: 6±1.7	Tissue NGS (total RNA)		- chronic active lesions were the most distinct from control WM based on the highest number of unique DEGs (n=2213), and differed the most from remyelinating lesions, indicating end of the spectrums in lesion evolution. - CD26/DPP4 was expressed by a subpopulation of microglia in the NAWM. - TGFβ-R2 was the central hub in the *de novo* network of common lesion DEGs, and it was expressed by astrocytes in remyelinating lesions.	
Konjevic Sabolek et al. (49)	Laser-microdissected target areas of CD8 and perforin in active MS lesions of 4 patients		NGS (mRNA) of cells communicating with CD8+ cells		- Communication between CD8+ T cells and mononuclear phagocyte cells expressing *CD163* and *CD11b*.	
Van der Poel et al. (50)	- 5 NAGM (occipital cortex), 10 NAWM (CC) of MS - 5 GM (occipital cortex), 11 WM (CC) of non-neurological disease	PMD: 6:06±0.018h (control) 9:17±0.18h (MS) RIN: 7.3±0.4, 7± 0.5 (control) 8.1±0.3, 6.3± 0.8 (MS)	NGS (mRNA) of isolated microglia		- Microglia show a clear region-specific profile between WM and GM. - Homeostatic profile of microglia was maintained in the normal appearing tissues (no changes in P2RY12, TMEM119). - Different regional transcriptional changes in MS microglia: microglia in NAWM had genes related to lipid metabolism; NAGM microglia had genes related to glycolysis and iron homeostasis.	
Voskuhl et al. (51)	5 MS patients and 5 controls with regions including corpus callosum, optic chiasm, internal capsule, hippocampus, frontal cortex, and parietal cortex	RIN: 5.1-8.3 (control) 6.1-8.7 (MS)	Tissue NGS (mRNA)		- Corpus callosum and optic chiasm were the most significantly affected CNS regions in MS. - Myelinating oligodendrocytes were the cell type most enriched with DEGs in MS.	
Chiricosta et al. (52)	Six different brain areas (corpus callosum, hippocampus, optic chiasm, internal capsule, frontal cortex and parietal cortex)from 5 MS and 5 controls (data from Voskuhl et al. 2019)	RIN: 5.1-8.3 (control) 6.1-8.7 (MS)	Tissue NGS (mRNA)		*HSPA1A, HSPA1B, HSPA7, HSPA6, HSPH1* and *HSPA4L*, encoding for HSP70s, are significantly upregulated in corpus callosum, hippocampus, internal capsule, optic chiasm, and frontal or parietal cortex, between healthy individuals and MS patients.	
Frisch et al. (53)	The MS Atlas of Elkjaer et al. (48)	PMD: 8-30h RIN: 6±1.7	Tissue NGS (total RNA)		*VLA-4* is highly expressed in active lesions in non-treated PMS patients.	
Rodríguez-Lorenzo et al. (54)	Choroid plexus samples from 6 PMS patients and 6 controls	PMD: 4.33-11h RIN: ≥ 6.5	NGS (mRNA)		- 17 genes increased in CP of PMS, here among the ncRNA, *HIF1A-AS2*. - Transcript alterations were related to hypoxic responses and secretion of neuroprotective peptides.	
Elkjaer et al. (55)	71 MS brain samples and 25 control WM samples from Elkjaer et al. (48)	PMD: 8-30h RIN: 6±1.7	Tissue NGS (total RNA)		2.73% of the transcripts mapped to HERV transcripts. Here among HERV-W and HERV-H transcripts located close to the MS genetic risk locus at chromosome 7 regions were uniquely expressed in MS lesions.	
Elkjaer et al. (55)	73 MS brain samples and 25 control WM samples from Elkjaer et al. (48)	PMD: 8-30h RIN: 6±1.7	Tissue NGS (total RNA)		*APOC1* was significantly increased in active MS lesions and *PTPRG* significantly increased in all WM MS brain tissue, while both encoding proteins were upregulated in the CSF of multiple MS subtypes.	
Manuel et al. (94)	- Isolated microglia from 10 MS NAWM and 11 controls from van der Poel et al. (50) - 7 chronic active perilesional MS NAWM and 10 controls [from Hendrickx et al. (33)]		NGS data from both tissue and microglia in NAWM and WM		- Cross dataset evaluation suggested MAPK and JAK/STAT3 pathways as potential drug targets in MS. - *CDK4, IFITM3, MAPK1 MAPK3, METTL12B* were enriched colocalized genes in *de novo* network. - Rubidomycin hydrochloride and zafirlukast were suggested as potential medications for drug repositioning strategies.	
**Single nucleus RNA next-generation sequencing (snRNA-seq) of tissue and isolated cells**						
**Authors**	**Patients**	**Quality** **(PMD, RIN)**	**Methodology**		**Key findings**	
Jakel et al. (56)	- 3 active, 3 chronic inactive, 4 chronic active, 3 NAWM, 2 remyelinating lesions from 4 progressive MS patients - 5WM from 5 controls	RIN: 4.04±.41	Tissue snRNA-seq		- Fewer nuclei from OPCs in all MS lesions and in NAWM compared to control. - The intermediate Oligo6 cells were highly reduced in MS. - Skewing in the subclusters of mature oligodendrocytes between MS and control tissue: the Oligo1 cluster was depleted in MS, whereas the Oligo2, Oligo3, Oligo5 and ImOLG clusters were enriched.	
Masuda et al. (57)	- 5 patients with early active multiple sclerosis - 5 from healthy brain tissue removed during surgery for epilepsy	–	snRNA-seq of isolated microglia		- Microglia in MS had downregulation of homeostatic signature: *TMEM119, CX3CR1, P2RY12 and SLC2A5.* - Microglia could be separated into subsets with specialized functions as APC function, matrix-remodelling function, dampen cytotoxic functions.	
Schirmer et al. (58)	- 12 MS tissue samples (entire tissue blocks including lesion and non-lesion GM and WM areas plus meningeal tissue) - 9 tissue samples from control individuals	PMD:6-27h RIN: 6.8-9.1	Tissue snRNA-seq		- CUX2+ excitatory neurons in cortical layers 2-3 were the cell type predominantly lost - WM astrocytes underwent broad transcriptional changes in the areas surrounding the lesion rim, such as upregulation of GFAP and CD44. -Microglia were dramatically increased in number in MS. - Myelinating oligodendrocytes at lesions had signatures of cell stress, iron accumulation and MHC class I presentation.	
Wheeler et al. (59)	CNS samples from 4 MS and 5 controls (included datasets from other scRNA-seq studies: cortical and cerebellar astrocytes from 20 MS and 28 controls)	RIN: 6.3±.80	Tissue snRNA-seq		- An expanded astrocyte population in MS vs control characterized by decreased NRF2 activation and increased MAFG activation, DNA methylation, GM-CSF signalling and pro- inflammatory pathways activity.	
Absinta et al. (60)	- 6 chronic active rim, 5 chronic inactive rim, 2 lesion core, 4 periplaque from 5 patients with progressive MS - 3 WM from 3 sex-matched controls	PMD: 6-12h	Tissue snRNA-seq		- High glial and immune cell diversity between lesion cores, active or inactive rim, and periplaque WM. Discovery of a lymphocyte-microglia-astrocyte axis with the key involvement of C1q in chronic active rim. - Two main microglia subsets identified: MIMS-foamy and MIMS-iron. Additionally, microglia signatures in MS overlap with neurodegenerative diseases suggesting similar mechanisms between primary and secondary degermation. - MIMS target genes were regulated by lymphocytes with the involvement of C1q, and C1q- blocking antibody gave a more homeostatic microglia phenotype.	

We highlight the main findings in eight sections based on tissue types: (i) brain regional differences; (ii) NAWM; (iii) NAGM; (iv) WM lesions; (v) GM lesions; (vi) cell-specific changes; (vii) non-human transcripts, (viii) databases.

#### 3.1.1 Brain Regional Differences

Corpus callosum and optic chiasm were the most significantly affected CNS regions in a study, and myelinating oligodendrocytes were most enriched with differentially expressed genes (51). Heat shock proteins were upregulated in all examined brain regions (*HSPA1A, HSPA1B, HSPA7, HSPA6, HSPH1, HSPA4L*) (52). Genes important in antigen-presentation, inflammation and hypoxia-induced responses were altered in the corpus callosum and optic chiasm (*TAPBP, IRF4, CTSB, CD79A*), while *STAT6* and *HLA-DRB5* were only increased in the optic chiasm. However, these regional differences may also reflect the presence of different cell types expressing different types of regional specific “housekeeping genes” with distinct physiological functions and purpose.

DNA methylation was altered, and RNA levels of DNA mehyltransferase were increased in MS hippocampus following demyelination (40). This study identified hypomethylation upstream of six genes including *ANKA*, a major regulator of CD40-CD40L, and hypermethylation upstream of ten genes e.g. *WDR81*, *NHLH2, PLCH1* involved in neuronal survival, synaptic density and memory.

In the choroid plexus (CP), 17 genes were significantly upregulated in progressive MS patients (54). These genes were related to hypoxia, neuroprotection and secretion (e.g. *CXCL2, LYVE1, SNHG15, MT1X*, non-coding *HIFA1-AS3*), while strong inflammatory reactions were absent.

#### 3.1.2 NAWM

**Figure 3:**
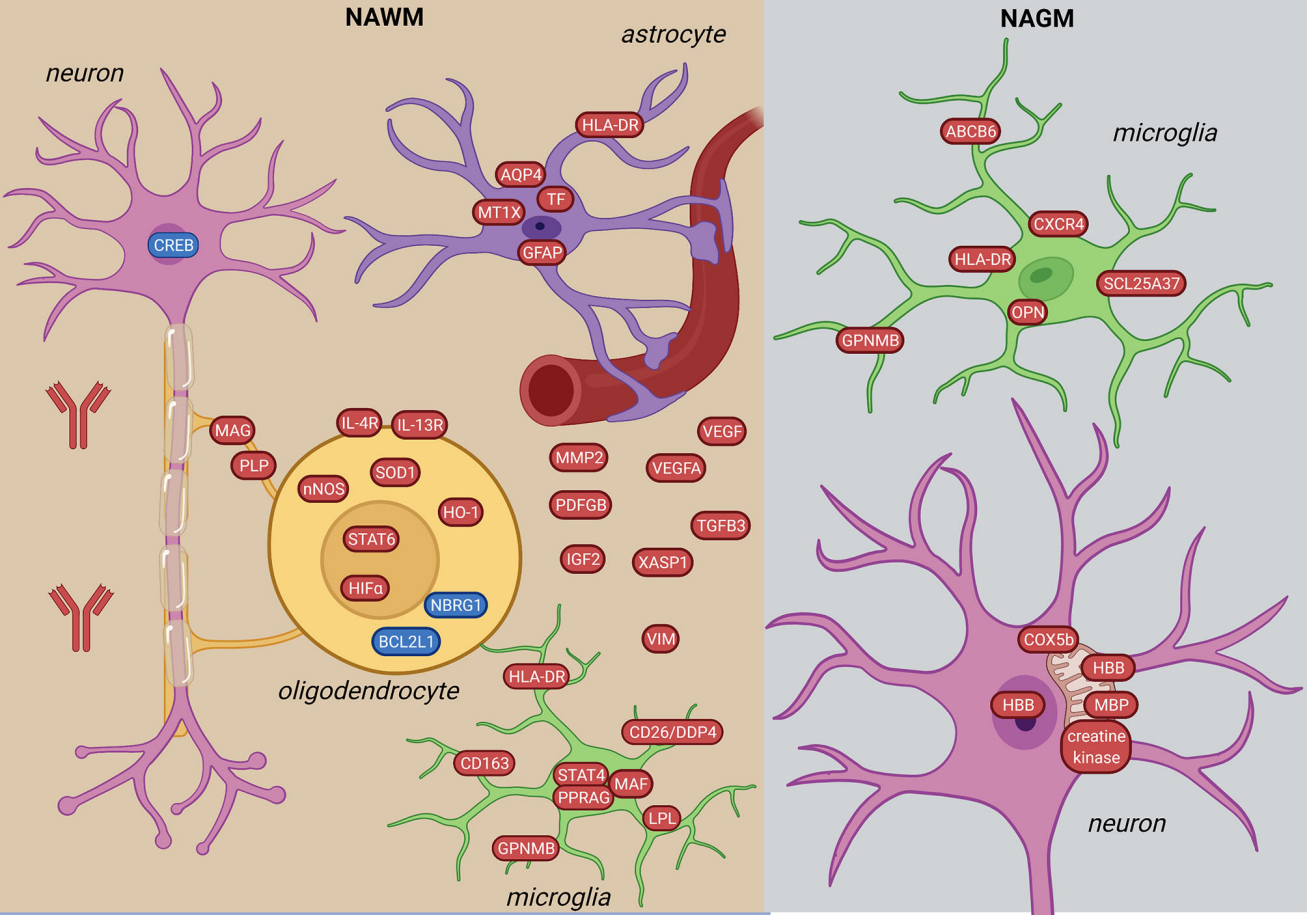
Signature of NAWM and NAGM in the MS brain based on transcriptome and proteome studies. In the NAWM, alterations in all brain resident cells were observed. Oligodendrocytes are characterized by altered myelin transcripts and upregulate anti-inflammatory and hypoxia-induced pathways (STAT6-, HIFα-signaling). Microglia upregulate pro-inflammatory molecules (STAT4-signaling, HLA-DR, GPNMB, CD163). Inflammatory astrocytes have iron- and oxidative stress-related profiles. In the NAGM, microglia have a distinct inflammation-induced neurodegenerative profile from NAWM (CXCR4, ABCB6, SCL25A37). Neurons in the NAGM express hemoglobin β (HBB) and have alterations in mitochondrial proteins. The figure was created by compiling data from several articles, and therefore molecules may not be expressed at the same time. Created with BioRender.com.

Comparing NAWM to control WM, 465 genes were differentially expressed (48). Among the top ten upregulated genes were immune-related (*IGHG1, HLA-DRB5, GPNMB, CD163*) and mitochondria-related (*MTRNR2L12, MTRNR2L8*). NAWM was also different from control WM by a global defense against oxidative stress based on upregulation of *STAT6, HIFα *and its target genes (21, 26, 61). Genes in the STAT-6 signaling were upregulated in oligodendrocytes (61) (**Figure 3**). These alterations were accompanied by upregulation of *nNOS, HO-1* and *HLA-DR*, suggesting an inflammatory and oxidative-stress related reaction in oligodendrocytes outside of lesions. A combined methylome and transcriptome study found downregulation and hypermethylation of oligodendrocyte survival genes in NAWM (*BCL2L2, NBRG1*) (46). Besides oligodendrocytes, several dysregulated genes in MS suggested alterations in subcortical WM neurons (21).

NAWM microglia upregulated *STAT4* and *HLA-DRα* (26), and had a lipid metabolic gene expression profile (e.g. *EEPD1, PPARG, LPL*) with unchanged expression of the homeostatic signature (*P2RY12* and *TMEM119*) (50). Additionally, a subtype of microglia (48) had increased expression of *CD26/DPP4* in the NAWM (46). Astrocytic markers (*GFAP*, *AQP4*) were also altered in the NAWM (61) (**Figure 3**).

Genes of several chemokines and cytokines (21, 26) were upregulated in NAWM reflecting the low level inflammation even without lesion formation. A mild disease course was also associated with a different molecular profile with altered expression of genes related to immune-regulation, myelination, anti-oxidative mechanism and neuroprotection together with a high hypothalamus-pituitary-adrenal (HPA) axis activity (35).

#### 3.1.3 NAGM

The difference in WM *vs*. GM microglia gene expression was significantly lower in MS compared to non-neurological disease brains. This suggests that microglia cells are losing region-specific profile in MS (50). However, while NAWM microglia have a lipid signature, NAGM microglia have increased expression of genes related to glycolysis and iron homeostasis (*SCL25A37, ABCB6*) and a neurodegenerative profile (*CXCR4, GPNMB, OPN/SPP1*) (**Figure 3**). Furthermore, in HLADRB1*15:01 positive patients, HLA-DRB1 and B5 were the highest expressed genes in NAGM (37).

#### 3.1.4 WM Lesions

**Figure 4:**
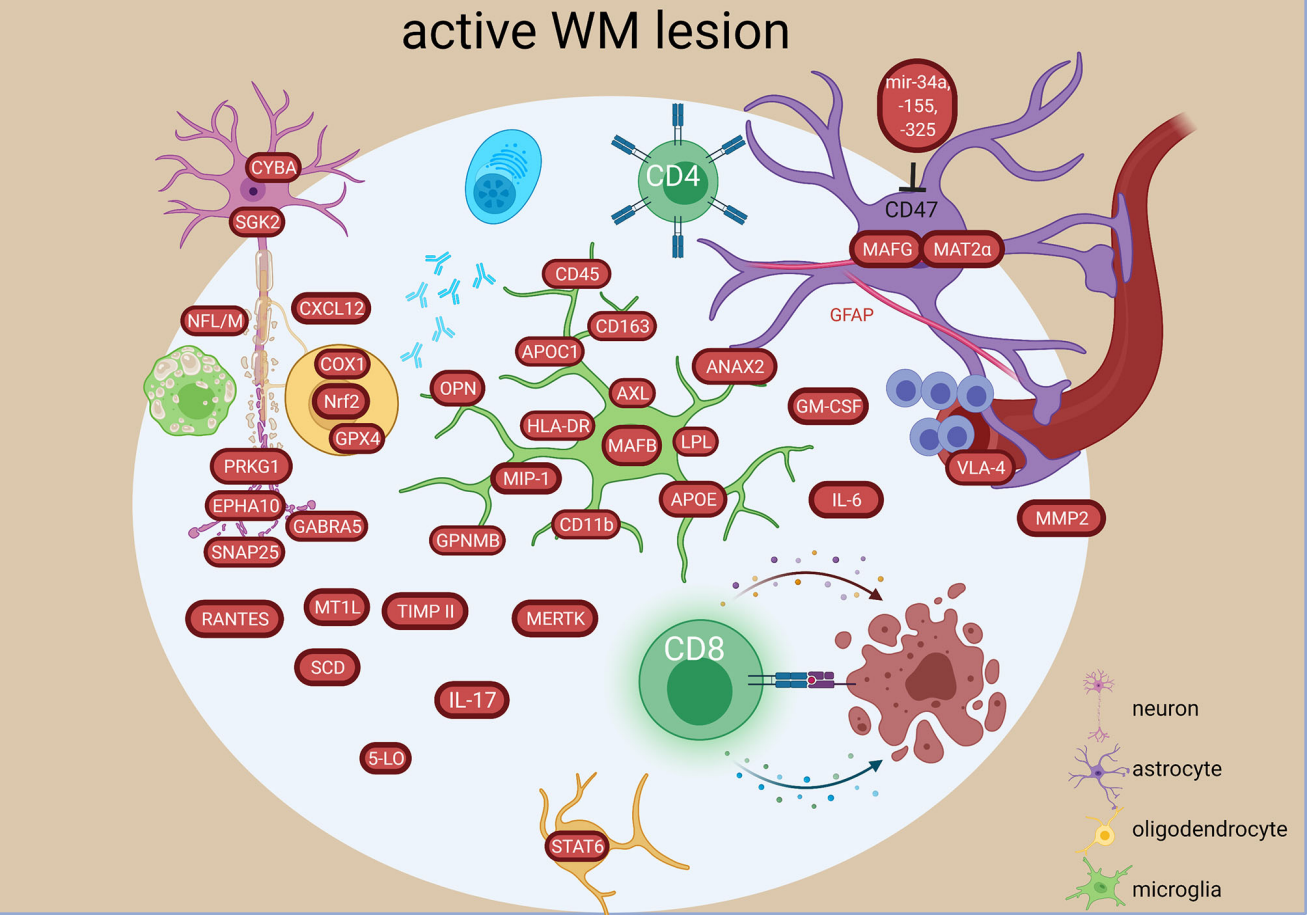
Signature of active WM lesion in the MS brain based on transcriptome and proteome studies. In the active lesion, an increase in both innate and adaptive inflammatory responses are present characterized by different molecular components in resident and infiltrating cells. An oxidative stress and degenerative profile especially in the oligodendrocytes and neurons have also been detected. The figure was created by compiling data from several articles, and therefore molecules may not be expressed at the same time. Created with BioRender.com.

A continuum of dysfunctional homeostasis (e.g. *VIM, HBB, MAF*) and inflammatory changes (e.g. *CASP1, IRF5, MMP2*) between active lesions and NAWM supports the concept of MS involving the whole CNS (24). However, the lesions differed from NAWM by high expression of genes related to immunoglobulin synthesis (*IGKC, IGL, IGGL1, ILR6*) and neuroglial differentiation (*SNAP25, CAP2, NFL/M*) (24). Upregulated genes in active lesions compared to NAWM also included chemokine genes and receptors (*MIP-a, RANTES, CCR1, CCR4, CCR5, VLA-4, CCR8*) genes, interferon- and tumor-necrosis factor receptors (17), and cytokines (*TGFB, IL-3, OPN, IL-5, IL6*) (18, 44, 53) (**Figure 4**). Two highly expressed genes encoded the Th cell marker (*CD4*) and the antigen-presenting gene (*HLA-DRa*) (18). Additionally, CD8+ T cells containing cytotoxic granules were suggested to communicate with mononuclear phagocyte cell expressing CD163 and CD11b in the lesions (49). Genes encoding multiple autoantigens were also found in MS lesions indicating a secondary autoimmune stimulation that could exacerbate the ongoing inflammation (43).

Mitochondrial injury in initial WM lesions was indicated by increase of *ND1-6, CYTB, COX1, CYBA, MPO, PTGS1, PXDN, GPX4, PRDX1, SGK2, ALOX12, EPHX2* expression, which were related to degeneration of oligodendrocytes and neurons and contributed to reactive oxygen species production by activated microglia and macrophages (28) (**Figure 4**).

Active and chronic active lesions shared upregulation of a number of genes coding for e.g. iron-binding protein (*TF*), chemokine and its receptor important for T cell accumulation in CNS (*CXCR3, CXCL10*), the myelin-binding protein (*MBP*), the first subcomponent of the complement system (*C1QB*), oxidative protection (*GPX1, SOD1*) and cytokines (*IL-6, IL-17, INFg*) (20, 23). However, 70 uniquely differentially expressed genes were also found: e.g. coding for the receptor related to differentiation (*EPHB6*), the granulocyte-macrophage colony-stimulating factor (*GM-CSF*), and a MHC class I molecule (*HLA-A*) in active lesions or e.g. genes coding for the chaperone protein (*HSPA1A*), component of MHC class I (*B2M*) or complement factor 4B (*C4B*) in chronic active lesions.

Differences have also been found on an epigenetic level, as the microRNA profile was different between active and inactive lesions (39). In the active lesions, microRNA-34a, -155 and -326 were all upregulated and targeted the *CD47* in brain resident cells to release inhibitor control and promote phagocytosis (**Figure 4**). Moreover, upregulated miR-22, miR-320 in active lesion and upregulated miR-30d in inactive lesions (39) were related to pathogenic changes (41), while downregulation of miR-18a, miR23b in inactive lesions (39) were related to protective changes correlating with MRI abnormalities (41).

**Figure 5:**
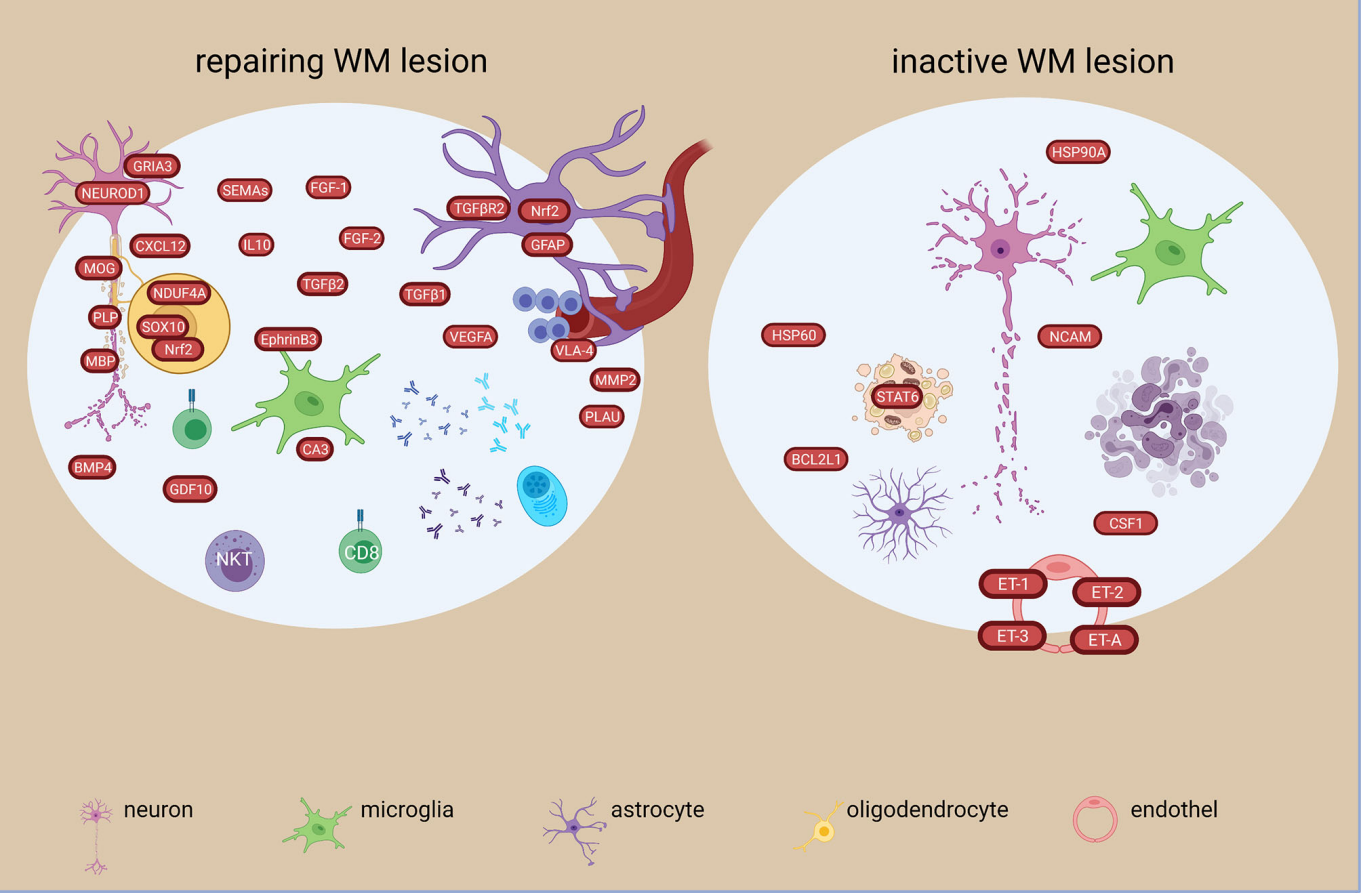
Signatures of repairing/remyelinating and inactive WM lesion types in the MS brain based on transcriptome and proteome studies. Remyelinating signatures are characterized among others by soluble growth factors and reparatory molecules such as FGF-1, -2, TGFB1,-2, BMP4 and GDF10. Oxidative and anti-oxidative responses are present, as well as a heterogenous immune response. In the inactive lesion, different heat shock proteins are present together with changes in endothelin transcripts. The figure was created by compiling data from several articles, and therefore molecules may not be expressed at the same time. Created with BioRender.com.

An in-depth investigation of different lesion types (active, early remyelinating, chronic active, inactive) in the WM showed extreme diverse events at transcriptome level. More differential expressed genes were unique than shared. Among the 282 altered genes common to all lesion types were genes related to inflammation (*STAT6, CXCL12, TNFs, DPP4/CD26, ITGA4, GPNMB, IL16, HLA-DRB5, MAFB, IGHG1, IGF2, MMP2*), phagocytosis (*SCD*, *CD163, MERTK*) complement pathway (*CFH, C7, CFI*), apoptosis/necroptosis (*FADS1, CASP1,-4*, *MLKL*) (48). Immunoglobulin genes were among the top 10 in all WM MS tissues, but the most heterogeneous expression pattern was detected in early remyelinating lesions. *TGFBR2* was the major molecular hub of the largest shared lesion network and was highly expressed in remyelinating lesions by astrocytes (48) (**Figure 4**). The most different signatures were found between remyelinating and chronic active lesions. Chronic active lesions had the highest number of unique genes reflecting intrinsic neuronal alterations, and *de novo* networks suggested an end-stage exhaustion (48). Most of the uniquely expressed genes in the early remyelinating lesions were non-coding RNAs, while others were related to lymphocytes and NKT cells (e.g. *CD8a, TIAM1, CTSW, CCL5/RANTES*), growth and development (e.g. *PEG10, BMP4, GDF10*), vascular changes and remodeling (e.g. *PLAU, VEGFA, CTGF*), mitochondria and protective stress responses (e.g. *NDUFA4, NOSTRIN*), lipid metabolism (e.g. *ACACA, ACOX2, ADH6, CA3*), and neurons (e.g. *NEUROD1, NLGN1, GRIA3*) (**Figure 5**). Another study found *CXCL12*, *SCD*, *STAT6* increased in all lesion types, and transcriptional differences between lesion types reflected a heterogeneous oligodendrogliogenesis (34). Quantitative changes of oligodendrocyte regulators were also found in remyelinating lesions (30). Compared to demyelinating lesions, remyelination was accompanied by significant changes in the expression of myelin proteins (*CNP, MAG, MBP, MOBP, MOG, OMG, PLP1)*, anti-inflammatory *IL10*, and semaphorins (*SEMA3C, SEMA4D, SEMA6A, SEMA6D, SEMA7A*) (**Figure 5**). The growth factor gene *FGF1* was significantly increased in remyelinating lesions compared to both control WM and demyelinating lesions. In functional experiments, FGF1 promoted both developmental myelination and remyelination by inducing LIF and CXCL8 in astrocytes to recruit oligodendrocytes. *GFAP* was also significantly increased in active and remyelinating lesions (55) (**Figures 4**, **5**). The glia receptor protein tyrosine phosphatase gene *PTPRG* was increased in all MS WM tissues, and was also significantly increased in the CSF of MS patients compared to healthy and other neurological disease controls (55). *CHI3L1* was increased in astrocytes in the chronic active lesion rim (55), and by microglia in active lesions compared to NAWM (50).

In a single-nucleus study of WM lesions, the majority of cells were oligodendrocytes, and oligodendrocytes represented the most heterogenous cell population (56). One of the seven oligodendrocyte populations was termed immune oligodendroglia (imOLG) due to expression of *APOE* and *CD74*. OPCs were reduced in lesions and NAWM compared to control WM. One oligodendrocyte population was depleted, whereas three others and imOLG were enriched in MS. Several myelin protein genes were upregulated in mature oligodendrocytes in MS, however some of those (e.g. *CNP, MAG*) were downregulated in remyelinating lesions.

Excessive expression of the antioxidant transcription factor *NRF2* in oligodendrocytes indicated oxidative stress and degeneration at sites of initial demyelination in active lesions (31). *NRF2* in astrocytes and macrophages were mainly seen in the later stages of active lesions with profound loss of oligodendrocytes. *NRF2* in neurons was low or absent despite NRF2-positve oligodendrocytes in close proximity indicating cellular differences in reaction to oxidative stress and inflammation (**Figure 4**).

**Figure 6:**
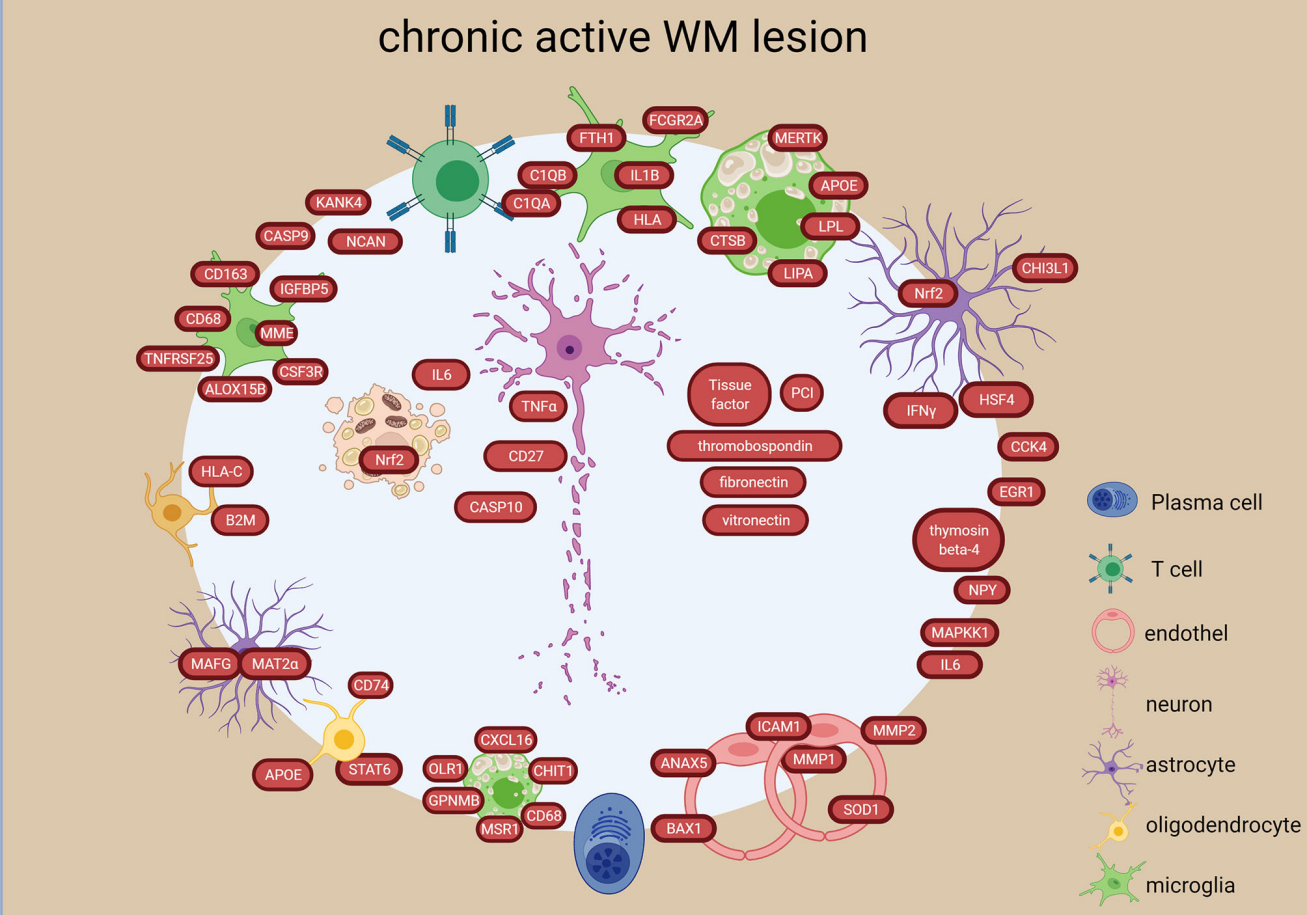
Signatures of chronic active lesion in the WM lesion types in the MS brain based on transcriptome and proteome studies. Chronic active lesion has a different molecular profile in rim *vs* center. Most activity is present in the rim with stressed astrocytes and oligodendroglia, proinflammatory microglial polarization and foamy macrophages. Additionally, presence of coagulation factors and endothelial alterations are detected. The chronic active lesion displayed the highest number of neuronal/axonal intracellular components. The figure was created by compiling data from several articles, and therefore molecules may not be expressed at the same time. Created with BioRender.com.

In chronic active lesions,14 genes were significantly upregulated in the rim *vs* the center (e.g. *IFNG, NGF2, CD4, CASP9, MAPKK1*) (22, 25, 29) (**Figure 6**). Inflammatory genes were upregulated in chronic active lesion center (*CCL4, IL6, CD27, TNFA*) (**Figure 6**), while upregulation of *NCAM, CSF1, HSP60, HSP90A, BCL2L1* in inactive lesion center and rim highlighted different inflammatory responses, beside apoptosis and stress (**Figure 5**). Heat shock protein genes in inactive lesions (48) and in the rim of chronic active lesions were upregulated, especially the heat shock factor 4 (HSF4) (29).

Upregulation of 165 genes and downregulation of 35 genes were identified in the chronic active lesion/slowly expanding lesions compared to inactive as well as NAWM (38). The upregulated genes suggested accumulation of microglia with proinflammatory differentiation at the lesion edge (e.g. *CD163, CD68, CSF3R, IGFBP5, ALOX15B, MME*, *TNFRSF25*) (**Figure 6**). A study that investigated the rim and peri-lesional regions of both chronic active and inactive lesions, found upregulation of previously not reported genes in the rim of chronic active lesions (*NPY, KANK4, NCAN, TKTL1, ANO4*) (33) (**Figure 6**). They also found that foamy macrophages in the rim upregulated genes involved in lipid binding and uptake indicating the expansion of demyelination (e.g. *MSR1, CD68, CXCL16, OLR1, CHIT1, GPNMB* all (**Figure 6**). Stressed oligodendrocytes with iron overload, reactive astrocytes and activated phagocytosing cells were also detected in the rim of chronic active lesions (58). These findings were confirmed and elaborated in a recent snRNA-study, where they found immunological-active OPCs, inflamed astrocytes (AIMS) and microglia (MIMS) in the chronic active rim (60). These were strongly connected to a high number of T cells and plasma cells suggesting an active role of the adaptive immune system in lesion expansion in collaboration with the glia cells in the smoldering inflammatory lesions (60). Microglia consisted of two distinct functional subtypes: the MIMS-foamy characterized by myelin phagocytosis and clearance properties, and the MIMS-iron, characterized by expression of complement C1q-complex, antigen-presentation and direct propagation of inflammatory damage at the lesion edge. The inflamed astrocytes were enriched for response to lipid, corticosteroids, wounding and expression of C3 similar to the A1 phenotype identified in the GM (62).

#### 3.1.5 GM Lesions

**Figure 7:**
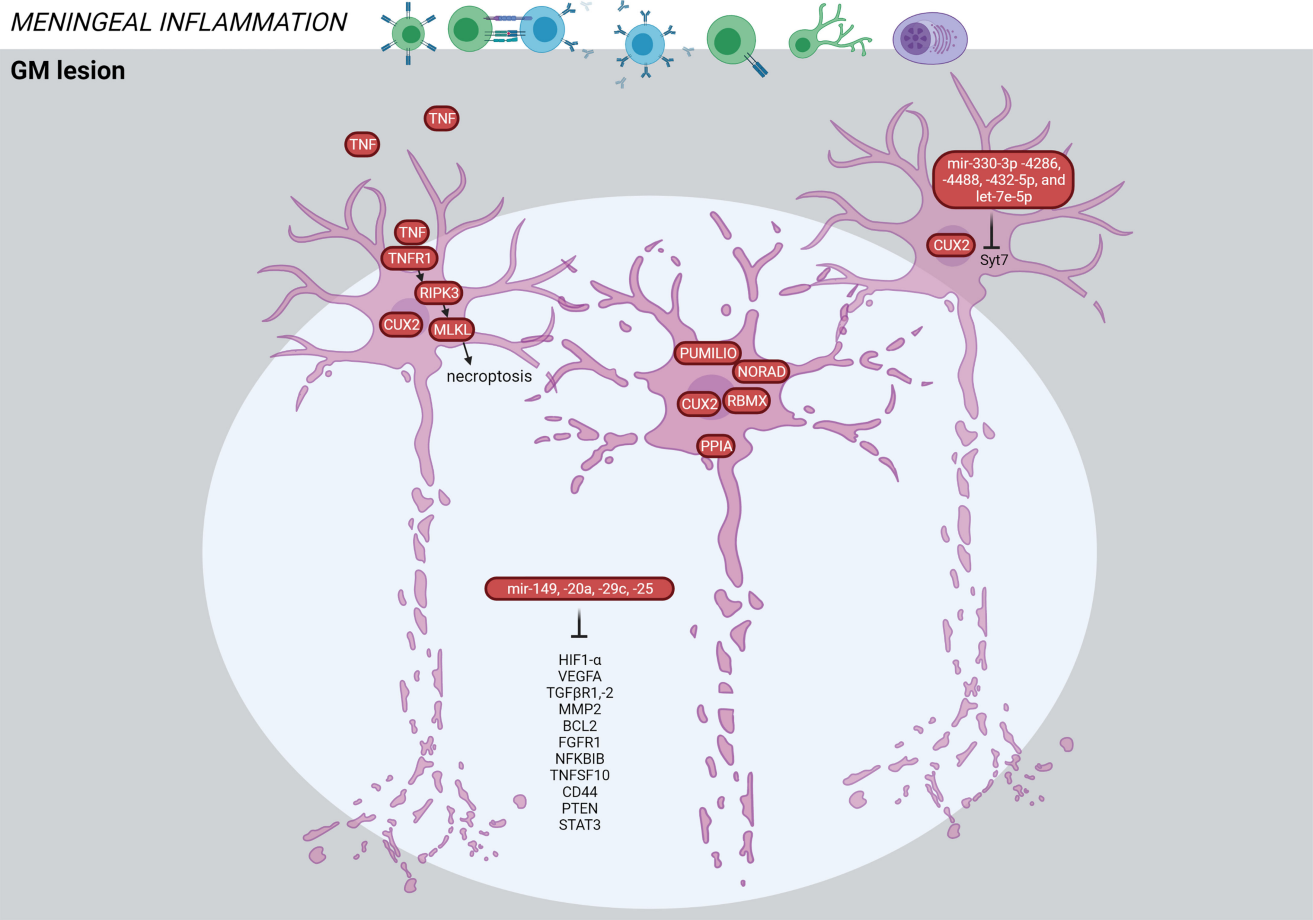
Signature of the GM lesion in the MS brain based on transcriptome and proteome studies. GM lesions are characterized by neuronal death mediated through TNF signaling. The CUX2-expressing cells are particularly vulnerable for degeneration. Alterations in microRNAs have been detected in the GM lesions associated with cortical atrophy. The figure was created by compiling data from several articles, and therefore molecules may not be expressed at the same time. Created with BioRender.com.

A combined microRNA and mRNA profiling in GM lesions *vs* NAGM found significantly regulated microRNAs in GM lesions, which target genes of axonal guidance, TGFβ-signaling and FOXO signaling (63). Out of 27 significantly altered microRNAs, four microRNAs (mir149, mir20a, mir29c, mir25) and their targets (e.g*. HIF1A, VEGFA, TGFBR1, TGFBR2, NFKBIB, FGFR1, TNFSF10, BCL2, MAP2K4, STAT3, MMP2, PTEN, CD44*) were associated with GM atrophy (**Figure 7**) (63). Three of the 27 significantly altered microRNAs were also detected in another GM lesional microarray study (mir181b, mir129-5p, mir1180) (64). Additionally, miR-330-3p, miR-4286, miR-4488, let-7e-5p and miR-432-5p shared the same mRNA target, the Syt7 gene coding for the neuroaxonal protein normally transported to synapses. These 5 microRNAs may be protective against Syt7 accumulation in the soma resulting in disturbed axonal transport.

TNF signaling was also significantly increased in GM lesions. Increased meningeal inflammation was associated with a shift from TNFR1/TNFR2 and NFkB-mediated anti-apoptotic pathways towards TNFR1- and RIPK3-mediated pro-apoptotic/pro-necroptotic signaling (36) (**Figure 7**). TNFR1 was expressed by neurons and oligodendrocytes, while TNFR2 was predominantly expressed by astrocytes and microglia. The authors suggest that immune cells in meninges generate a milieu of increased demyelination and neurodegeneration by changing the balance of TNF signaling.

Another study found a selective loss of neurons expressing the transcription factor *CUX2* in upper cortical layer lesions associated with pronounced meningeal B cell infiltration (58). These neurons expressed markers of cellular stress (*PPIA, NORAD, PUMILIO, RBMX*), and their loss may be a key event in MS progression and cortical atrophy (**Figure 7**).

#### 3.1.6 Cell-Specific Changes

A study focused on endothelial cells in vessels found 52 genes significantly altered in chronic active or inactive lesions compared to control WM or NAWM (27). The majority of these genes belonged to endothelial cell activation, while *VEGFA* was the only one belonging to angiogenesis. Most of the genes were highly expressed in chronic active lesions compared to control WM (*ANXA5, CSF3, FGF1,-2, FLT1,-4, ICAM1, MMP1, -2*) (**Figure 6**) and compared to NAWM (*FGF2, FLT1,-3, MMP14, PLAU, RIPK1*). Several endothelin genes (*1,2,3,A*) involved in constriction of blood vessels and supply were increased in inactive lesions compared to NAWM (**Figure 5**).

Transcriptional profiling of isolated astrocytes in NAWM also revealed increased gene expression related to iron metabolism, oxidative stress, and inflammatory response (32) (**Figure 3**). An astrocyte single-cell study identified an expanded astrocyte population in active lesions characterized by decreased *NRF2* and increased *MAFG*, GM-CSF signaling, pro-inflammatory pathway activity and DNA methylation (*DNMT1*) (59) (**Figure 4**). This astrocyte population is characterized by a MAFG/MAT2α-driven pro-inflammatory genomic program contributing to the pathology and may be induced by GM-CSF produced by infiltrating T cells (**Figure 4**). This corresponds to the high *GM-CSF* in active lesions (23), and low *NRF2* in astrocytes in initial demyelinating lesions (31).

**Figure 8:**
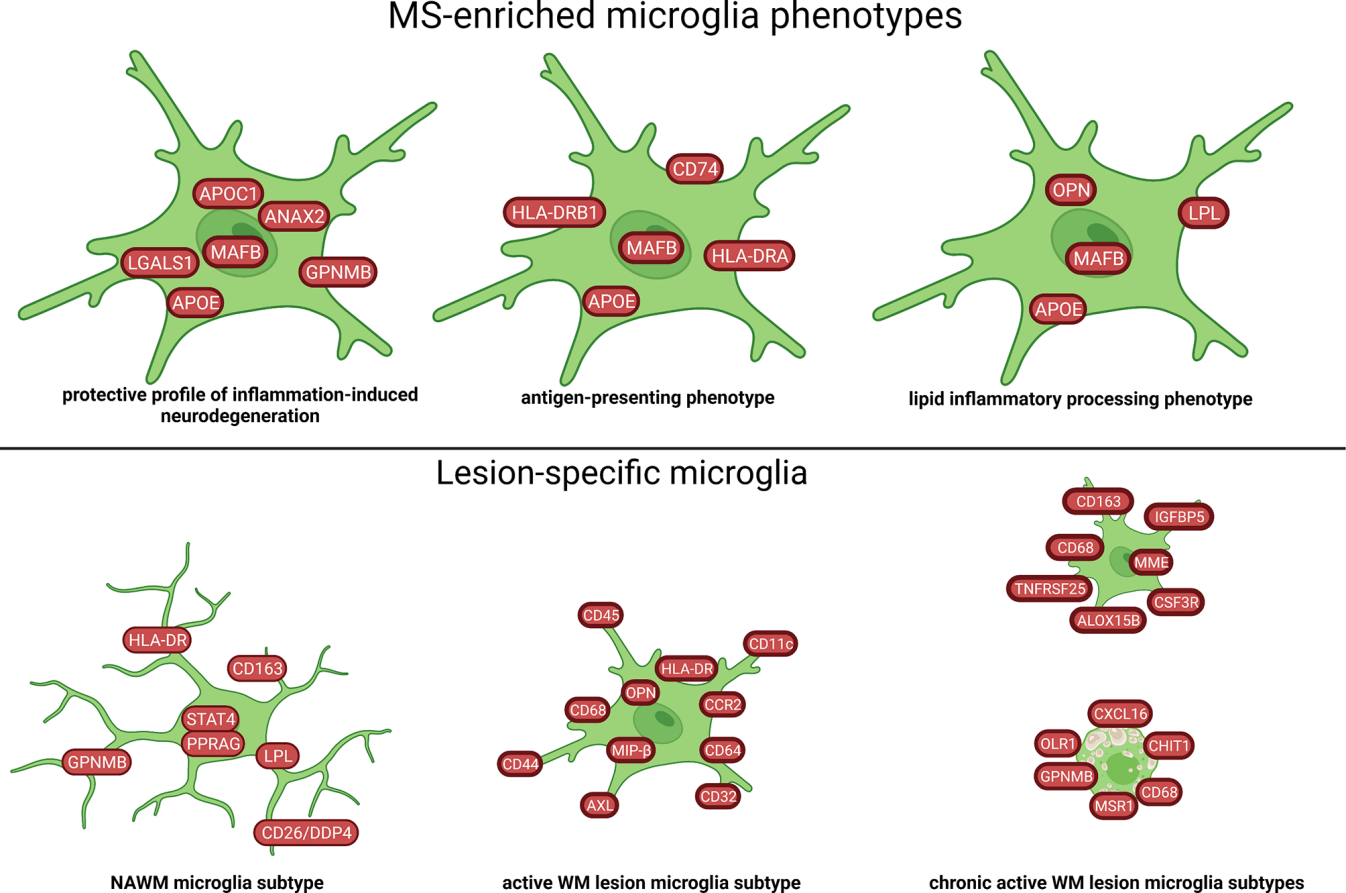
Signature of microglia subtypes in the WM of MS brain based on transcriptome and proteome studies. Different profiles of microglia in WM tissue of MS have been identified. The NAWM microglia subtype in the figure was created by compiling data from several articles, and therefore molecules may not be expressed at the same time. Created with BioRender.com.

Seven microglia cell populations expressing the core microglial genes (*TMEM119, P2RY12*) in the WM were discovered in a single-cell study (57). Two of these clusters were enriched in brains of MS patients and one was associated with MS. These three populations had increased levels of *APOE* and *MAFB* (**Figures 4**, **8**). The MS-associated microglia subset highly expressed *CTSD, APOC1, GPNMB, ANAX2, LGALS1*, while the two MS-enriched clusters showed high expression of either *CD74, HLA-DRA, HLA-DRB1* or *OPN*/*SPP1, PADI2, LPL* (**Figure 8**). These findings suggest distinct disease-related subtypes of microglia in the MS brain, which were similar to microglia subtypes in a demyelination model. However, subsets of microglia varied substantially between individual patients indicating high inter-individual heterogeneity. Additionally, the different microglia populations appeared as a transcriptional continuum of the local populations, which could reflect the ability of microglia to easily adapt to changes in the surroundings.

Methylome changes within neuronal nuclei in WM suggested alterations in axonal guidance, synaptic plasticity and CREB signaling in MS (42). The CREB activity was reduced in NAWM compared to WM neurons suggesting alteration of CREB signaling prior focal tissue damage (**Figure 3**). Neurons from MS patients displayed epigenetic alterations affecting several genes of the glutamate/GABA signaling along with interconnected cellular networks (semaphoring/plexin, Slit/ROBO, Shh/Wnt signaling). Lesion-associated changes in genes implicated neuronal projections and synaptic processes (e.g. *GABRA5, PRKG1, DLGAP3/SAPAP3*) (42) (**Figure 4**).

#### 3.1.7 Non-Human Transcripts

Amplicon next-generation sequencing of the human endogenous retrovirus (HERV)-W group found very similar transcript level between of WM lesions and control WM but evidence for interindividual differences in HERV-W transcript levels (45). In another study, genome-wide HERVs expression level between MS WM and control WM was not different (65). However, transcripts of HERV-W were reduced in chronic active and repairing lesions. Additionally, four different transcripts of HERV-W on chromosome 7 were only present in the MS brain (65). Another study found HERVs significantly overexpressed in demyelinating brain tissue including several retroviral domains (core, envelope, integrase, reverse transcriptase) (47). However, the overexpression was small. Due to multiple similar HERV transcripts incorporated and spread out throughout the human genome, examination of them is difficult.

Presence of microbial RNA sequences and bacterial antigens were associated with demyelinating brain lesions (66). In the study, they found 29 MS microbial candidate genera from 11 different phyla, most of which were anaerobic.

#### 3.1.8 Databases

Based on these transcriptomics data, novel interactive online databases were generated. The MS Atlas (www.msatlas.dk), comprises comprehensive high-quality transcriptomic profiles of 98 different WM lesion types (53). The user-friendly MS Atlas was designed to provide information about significant expression of candidate genes and their participation in *de novo* protein-protein interaction networks in different MS lesions (53, 67). The OligoInternode database (https://ki.se/en/mbb/oligointernode), and the single cell expression atlas (https://www.ebi.ac.uk/gxa/sc/experiments/E-HCAD-35/results/tsne) give information about gene expression from single cells in MS lesions.

### 3.2 Systems Proteomics to Examine Pathological Mechanisms in the MS Brain Tissue

Proteomics has also been developed as a large-scale unbiased tool for identifying final products of cells and post-translational modifications such as phosphorylation, glycosylation and acetylation associated with MS (68, 69). Despite various proteome studies in brains of animal models of MS, only a few proteome studies of MS CNS tissue have been performed (**Table 3**).

**Table 3:** An overview of the studies (n=11) that examined the proteome profile in human MS brain tissue.

**Authors**	**Patients**	**Quality** **(PMD)**	**Methodology**	**Key findings**
Newcombe et al. (70)	- 3 WM lesions and adjacent NAWM from 3 blocks of 1 MS patient - 3 blocks of control CM from 2 controls	PMD: 8-15h	LC-MS/MS (MALDI-ToF) with reduction of abundant cytoskeletal proteins	- Cluster analysis based on 109 proteins showed three clusters: WM, NAWM and lesion. - WM samples or lesion samples could cluster with NAWM, but MS lesion and WM samples did not cluster together
Han et al. (71)	- 2 Active, 2 chronic active and 2 chronic lesions of fresh-frozen from 6 MS patients - Normal WM from 2 controls	PMD: 4-24h	LCM, LC-MS/MS (ESI)	- Number of unique proteins in the major lesion types: 158 for active, 416 for chronic active and 236 for chronic lesions. - Revealed 5 proteins involved in coagulation unique for chronic active lesions: tissue factor, PCI, thrombospondin, fibronectin and vitronectin.
Fissolo et al. (72)	- 8 samples from 8 MS patients	PMD: 8-38h	LC-MS/MS (ESI) with antibodies against HLAs	- Identified processed peptides presented on MHC I and II molecules from MS brains as self-antigens of diverse MBP peptides as well as GFAP, NFL, APOD, APOE, ferritin, transferrin - By characterizing the MHC ligandome of MS brain tissue, they identified 118 amino acid sequences from self-proteins from MHC I and 191 from MHC II molecules.
Ly et al. (73)	- 12 chronic active lesions, 8 chronic periplaque WM (PPWM), 12 late reyelinating lesions (LRM), 11 LRM PPWM from 3 MS patients (areas within same category were pooled within patient samples) - 6 WM areas from 4 controls	PMD: 8-58h	LCM, LC-MS/MS (ESI) with iTRAQ labelling	- Myelin-associated glycoprotein was significantly downregulated in chronic demyelinated lesions compared to late remyelinated lesions, NAWM and WM. - The number of protein identifications obtained from chronic lesions was significantly higher than in all other lesional/NAWM areas. - Contactin was downregulated in the NAWM surrounding chronic lesions compared to WM. - GFAP was upregulated in chronic lesions compared to NAWM and DWM. - HAPLN2 was downregulated in late remyelinated lesions and NAWM vs WM. - Upregulation of PRX-6 in chronic lesions vs chronic NAWM.
Broadwater et al. (74)	- parietal, Brodmann areas 1-3, frontal cortex and Brodmann area from 8 MS brains and 8 control brains	PMD: 3-30h	LC-MS/MS (SELDI-ToF)	- 4 proteins differentially expressed: COX5b, brain specific creatine kinase, hemoglobin-b-chain and MBP.
Brown et al. (75)	- 5 postmortem cortical MS tissue - 5 cortical areas from control brains	PMD: 3-23h	LC-MS/MS (ESI)	- 15 proteins including hemoglobin β subunit (Hbb) were identified. - Hbb was enriched in pyramidal neurons in internal layers of the cortex, and interacted with subunits of ATP synthase, histones, and a histone lysine demethylase.
Syed et al. (76)	- 3 chronic active, 3 active lesions, 2 peri-lesional WM and 1 NAWM from MS	PMD: 7-22h	LCM, LC-MS/MS (ESI)	- Ephrin3, an oligodendrocyte differentiation inhibitor, was expressed in demyelinated WM lesions.
Maccarrone et al. (77)	Discovery cohort: - NAWM, NAGM, and lesions with different extent of remyelination from 1 SPMS Validation cohort: - 12 PMS blocks	PMD: 8-24h	MALDI-IMS LC-MS/MS (ESI)	- Lesions with low remyelination had compounds of molecular weights smaller than 5300 Da, whereas completely remyelination had molecular weights of more than 15200 Da. - Tymosin beta-4 was highly expressed in demyelinated lesion rim.
Qendro et al. (68)	- brain lesions of 2 acute MS patients	PMD: 4-24h	LC-MS/MS (ESI) Peptide microarray Exom sequencing	- Mutated forms of proteolipid protein 1 (PLP1).
Faigle et al. (78)	- GM samples from 6 controls and 6 MS cases, WM samples from 3 controls and 9 MS cases.	PMD: 5-22h	LC-MS/MS (ESI)	- Identification of novel citrullinated peptides and already described citrullinated proteins: MBP, GFAP, and vimentin. - Modified proteins in MS WM was higher than control tissue and increased citrullination in WM compared to GM.
Böttcher et al. (79)	10 WM lesions and 10 NAWM from PMS 8 WM from controls	PMD: 4:21-10:20h	Single-cell mass cytometry with CyTOF of isolated microglia	- decreased abundance of homeostatic microglial markers, while increased expression of APC-, phagocytosis-, inflammatory- and apoptosis-related markers in active lesions. - TNFhi microglial cluster was higher in NAWM compared to active lesion - monocyte-derived macrophages were scarce in active lesions

#### 3.2.1 WM Immune Activity

A proteome study found that 109 proteins could separate WM lesions from adjacent NAWM and control WM (70). Overlap was only observed between NAWM and WM lesions, but not between NAWM and control WM.

To characterize the MHC-bound peptide repertoire in MS brains, proteomics was performed on captured HLA-A, B, C, and DRs. 118 amino acid sequences from MHC I and 191 from MHC II were eluted corresponding to 174 identified proteins including both known and novel autoantigens (72). Some were involved in apoptosis (annexin A1, BCL2-associated TF1), enzymes (GDH, GS, G3PD, NADH dehydrogenase), cytoskeleton (actin, α-ubulin), immune responses (CXCR1, IL12R), CNS structure (NFL, GFAP, MBP, α-synuclein), and serum proteins/iron-related/coagulation (APOD, APOE, ferritin, transferrin, von Willebrand factor). These proteins within the MHC ligandome mirror the proteins involved in different features of the MS pathology.

Combined proteomics and genomics on two acute MS autopsied brain samples detected seven unique mutations of PLP1 (68). This was confirmed with in-depth genomic analysis on mRNA, but not in the genomic DNA, highlighting how results from integrative approaches can strengthen the discovery of specific and precise pathogenic mechanisms in MS.

Myeloid cells from active lesions, NAWM and WM in progressive MS (PMS) were analyzed using single-cell mass cytometry and found lower abundance of microglial homeostatic proteins in active lesions (P2Y12, TMEM119, CXC3R1, GPR56) (79). The myeloid cells in the active lesions were highly phagocytotic and activated indicated by upregulation of CD45, HLADR, CD44, CD114, CD11c, CD68, MS4A4A, CCR2, CD64, CD32, AXL, NFAT1, CD95, Clec7a, CD47, MIP-1β (CCL4) OPN (SPP1) (**Figure 4**). However, infiltrating myeloid cells were scarce in active lesions in PMS. Additionally, the TNF^hi^ microglia population was reduced in active lesions compared to NAWM.

#### 3.2.2 Two Proteins Important in Remyelination?

Unsupervised clustering of proteomics data led to discovery of cortical lesions, which were not detectable by routine histology (77). They identified tymosin beta-4 mainly expressed in macrophages and activated microglia at the rim of chronic active WM lesions and in the GM (**Figure 6**). Tymosin beta-4 is involved in neurite extension and plays a role in restoring and remodeling neurons and in remyelination.

Another study found upregulation of the receptor tyrosine kinase Ephrin3 in the MS lesions. Tissue extracts from MS lesions inhibited OPC, while antibody-mediated masking of EphrinB3 epitopes promoted it (76) (**Figure 5**). These proteomics studies suggest that EphrinB3 and tymosin beta-4 may be potential targets to promote remyelination.

#### 3.2.3 Coagulation and Hemoglobin β

Proteomics of microdissected active, chronic active and inactive lesions showed that chronic active lesions displayed the highest number of uniquely dysregulated proteins, and proteins of unknown function made up more than half of the unique proteins (71). This was supported by an independent study in 2011 (73). Five proteins involved in coagulation were unique to chronic active lesions (tissue factor, PCI, thromobospondin, fibronectin, vitronectin) (71) (**Figure 6**). Coagulation factors in the CNS interfere with synaptic homeostasis and neuronal networks, and act pleiotropic on different receptors of both resident and circulating cells as well as the extracellular matrix (80).

Another study found dysregulated proteins associated with extracellular matrix, oxidative stress and myelin sheath (73). There was decreased abundance of MAG (oligodendrocytes) and contactin-1 (neurons), while increase in GFAP (astrocytes) in the chronic active lesions in a milieu with abundant anti-oxidant PRX6 and metabolic processes (alfa-enolase).

Proteome studies with co-immunoprecipitation have discovered that hemoglobin β may play a role in neuronal energetics by interacting with histones in the nucleus and by binding to proteins in mitochondria (74, 75) (**Figure 7**).

#### 3.2.4 Post-Translational Protein Modifications – A Missing Link

Studies on post-translational modifications will be the next layer of valuable information. Recently, a comprehensive analysis of citrullinated peptides in WM and GM of MS patients identified novel citrullinated sites of MBP, GFAP and vimentin, but their functional role remains unknown (78).

## 4 Discussion

Omics studies of MS brain tissue in the last four decades support MS as a global brain disease with inflammation, iron-disturbances, cellular-stress and hypoxia. However, some regions are more affected than others and the biggest transcriptional changes were detected in the corpus callosum and the optic chiasm (51). While microglia seem to lose the regional specificity in MS, there are similarities between MS microglia phenotypes and the microglia phenotypes during de- and remyelination in the cuprizone model, which also affects mainly the corpus callosum (57, 81). The most affected cell type seems to be oligodendrocyte (30, 34, 51, 56). This may not be surprising as the disease is characterized by demyelination. However, there is a bias towards a higher number of studies investigating the WM than GM. Considering the altered genes, the cell type may be more important than the tissue location, although the local environment, architecture and milieu may continuously drive the cell types into different phenotypic and functional subsets to adapt to the local surroundings.

Molecular components of TGFβ signaling and CREB signaling are altered in addition to multiple changes in semaphorin-, heat shock-, myelin-, APO- and especially multiple types of HLA-transcripts/proteins. Key differentially expressed molecules found multiple times independent of lesion stage are related to inflammatory responses (CD163, OPN, GPNMB, MIP-α/β), lipid metabolism (SCD, LPL, SOD1) cell trafficking (MMP2, CXCL12, VEGFA), but there has been bias in the selection of the examined tissue/cell types.

### 4.1 Oligodendrocytes

Even in the NAWM, oligodendrocytes have a different molecular profile similar to a survival mode against virtual hypoxia. They upregulate the hypoxia induced HIFα-signaling pathway and the STAT6-signaling pathway, which is associated with anti-inflammatory IL-4 and IL-13 receptor expression (21, 26, 46). The STAT6-signaling seems to be even more increased in oligodendrocytes in all lesion types (34, 48). However, there is a heterogeneity of oligodendrocyte subtypes between different lesion types, where even an immunological phenotype appears (56, 58). This immunophenotypic OPC was also seen at the rim of chronic active lesions (60).

Myelin proteins are altered in all studies including even the GM mitochondrial proteome (74). Nevertheless, different studies showed contradicting results: myelin transcripts and proteins can be reduced in remyelinating lesions (34, 48), while others found them upregulated (30, 76). This could be due to the different stages of remyelinating and remodeling processes captured by omics studies as static snapshots. Understanding the molecular mechanisms in remyelinating lesion using omics may be complicated, as non-coding RNAs dominate and no known predefined pathways have been found (34, 48), but for OPC differentiation FGF1-signaling through astrocytes, EphrinB3 and thymosin beta-4 may be important (30, 76, 82). Mapping the genetic programs of OPC and oligodendrocyte development/polarization in MS may help to unlock and even direct the remyelination process.

### 4.2 Microglia

Microglia play a role during all stages of lesion evolution in both the GM and WM. Even far from lesions, there are highly activated distinct microglia subtypes (26, 48, 50). This suggests an early activation of their local function, most probably cleanup, which may have been catalyzed by low level of chemokines and cytokines detected throughout the brain. In active lesions, the microglia profile is highly activated, and seems to be the dominated by signal transduction (*CD45)*, immunomodulation (*OPN, CD11*), antigen-presentation (*HLADR*) and phagocytotic properties *(AXL, CD68, CD163*) (79). The MS microglia expressing *APOE* and *MAFB* were divided into three subgroups: a protective profile of inflammation-induced neurodegeneration, an antigen-presenting phenotype and an inflammatory lipid-processing phenotype (57). However, there was a decrease in the TNF^high^ microglia subgroup in active lesion compared to NAWM (79). In the rim of the chronic active lesions, microglia may have a damaging *vs* repairing functional phenotype, and by mapping the interactome, microglia strongly interacted with immune cells with involvement of the C1q providing evidence for a lymphocyte-glia axis of lesion progression (60).

### 4.3 Astrocytes

Being the most abundant cells in the CNS, astrocytes also have altered phenotypes in MS with spatial molecular differences (58). Astrocytes have multiple key functions depending on the surrounding cells and tissue architecture (83). In the NAWM, astrocytes express transcripts associated with iron homeostasis, oxidative stress and immune-related genes (32). *GFAP* is also increased in remyelinating WM lesions (30, 55). In the GM, astrocytes upregulate the *NRF2* and its anti-oxidant target molecules, implying a reparatory and neuroprotective effect (31). However, a pathogenic pro-inflammatory subtype of astrocytes has also been detected and is characterized by reduced expression of *NRF2* and increased expression of *MAFG/MAT2α.* In the chronic active rim, reactive and inflamed astrocytes (AIMS) were detected expressing C3 and an A1-proinflammatory profile and in close interaction with the inflammatory microglia (60). This suggests that astrocytes can polarize to very distinct activation states, which are either damaging or beneficial in the MS pathogenesis. A detailed description of processes towards astrocytic polarization and functional changes are needed, as they can promote brain repair.

### 4.4 Neurons

Neuronal pathology and axonal injury are hallmarks of MS and major contributors to progression and permanent disability. Neurons in the NAWM have altered expression of genes involved in axonal and synaptic guidance as well as the CREB-mediated neuroprotective signaling pathway (42). NFL and α-synuclein as autoantigens also suggest direct immune attack against neurons (72).

In the GM tissue, TNF signaling seem to play a crucial role, where released TNF binds to TNFR1 on neurons and oligodendrocytes and activates pro-apoptotic/pro-necroptotic pathways leading to brain atrophy (36, 84). *CUX2*-expressing neurons in the upper cortical layers are most vulnerable for cell stress and death (58). Hemoglobin β in the MS neurons works as an epigenetic regulator and interacts with mitochondrial proteins, both ultimately controlling the energy metabolism (75).

### 4.5 The Mystery of the Chronic Active Rim

The number of chronic active lesions is increased in the progressive phase and is associated with aggressive disease course and poor clinical prognosis (85). However, it is unclear if the active rim purely expands the lesion, or it represents a cellular/molecular wall to halt progression, or even a battle in between. Moreover, data suggest that even though chronic active lesions are histologically similar, there may be differences on a genomic programming level. As snapshots, omics studies cannot answer if such differences represent distinct molecular mechanisms leading to lesion evolution or rather halting those. Based on multiple transcriptome and proteome studies, chronic active lesion is the most unique WM lesion type: it has the highest number of differentially regulated genes and proteins that may represent end-stage exhaustion, and it differs the most from control WM on molecular levels (48, 71, 73). Some of the unique proteins in chronic active lesions are involved in anti-oxidation and coagulation (71, 73), while many of the transcripts are neuronal/axonal (48). The uniqueness of chronic active lesions has also been identified by distinct and diverse cell populations connected through a lymphocyte-microglia-astrocyte axis that may be responsible for the smoldering inflammation (60).

### 4.6 Unbalanced Rate of Discovery Research *vs* Functional Research

Omics studies of tissue alone are very unlikely to lead to new treatments. However, the rate for finding differentially expressed transcripts/proteins and molecular networks is much faster than establishing their functional roles in a specific cell and in a given context. Thus, interpretation can end up with crude functional annotations, and therefore may even confuse results. Interpretation of omics in MS is often annotated to immune cells or immunological properties, even though molecules may have different functions in the brain depending on cell type. Therefore, functional experiments can enhance the interpretation of omics findings in the context of CNS.

### 4.7 Limitations, Considerations, and Recommendations of Multi-Omics

At least four main problems need to be solved: (i) sample size and quality, (ii) the “snapshot” characteristics of omics (iii) analytic obstacles, integration and gaps of data, (iv) relationship between clinical/pathological classification and tissue systems biology (endophenotypes).

#### 4.7.1 Quality

Sample size is often low due to high experimental cost, the need of specific laboratory equipment, and limited access to human MS brain tissue. Most studies conducted on brain tissue include a restricted number of patients, and overlapping these studies is also complicated due to inter-individual and inter-study variations. Additionally, availability of tissue from the early timeframe of the disease course or from the transition to progression is largely missing. Autopsy brain tissues often represent advanced stages of disease from older patients, while biopsy brain tissue is very limited, taken from specific sites and most often from patients with atypical MS. The postmortem delay of tissue varies considerably even within the same study (**Tables 2**, **3**). In transcriptomic studies, the RNA integrity number (RIN) value is often not mentioned, but the threshold for integrity also depends on which downstream approach is used (**Tables 2**, **3**). Qualities and quantities also differ, where most identified proteins are the highly abundant (86, 87), and low abundant proteins, likely to be involved in the distinct specific processes, remain to be discovered. Consequently, to find the true pathological signatures, reproducible and robust results are needed generated by well-designed studies including sample size power calculation, standardization of experimental as well as computational pipelines and independent validation. Furthermore, the high experimental costs and limited material demand consortiums and larger studies in collaborations across disciplines and nations using experimental and computational consensus pipelines. This kind of international network of MS experts have already begun as with the “Mystery Solved Project” (88).

#### 4.7.2 “Snapshot”

Omics provides only static snapshots of cells at different states in a limited area: only a moment is captured of the highly dynamic variations derived from the cell state kinetics, daily biological rhythms and even stratification of patient populations over time. Longitudinal studies or individual cell trajectory tools might be helpful, but the same cell can only be measured once. To overcome this, increasing the data size by learning a latent factor model would be necessary, which encodes some unknown cell state coupled with the cell type for deconvolution. This leads to another problem, where the rapidly produced comprehensive omics data challenge the current computational methods and tools for integrative analyses.

#### 4.7.3 Analytic Obstacles, Integration and Gaps of Data

There is a danger that too much trust is given to the output data without comprehending, how those data were obtained. Especially, there is no criteria for the sample size, the quality and standardized computational pipelines. Difficulties in combining different datasets have also been emphasized by a comparison of proteome, mRNA and protein abundance profiles of oligodendrocytes and myelin (89). The challenges to develop true robust integrative methods include different modalities, batch effects between experiments, low sequencing depth and high-modality interactions.

Furthermore, directly translate changes in the transcriptome to the dysregulated proteome is improper due to posttranscriptional regulations and spatial and temporal differences in the production of RNA and proteins. On top of that, protein function and turnover are intensely regulated by posttranslational modifications. Phosphorylation and cysteine modifications regulate protein activity; glycosylation affects protein-protein interactions; and ubiquitination affects protein localization and turnover. Activity of a protein, and its abundance in a cell cannot be deduced with certainty from the level of the corresponding mRNA.

Another challenge is to clarify, how single features are associated through multiple interactions across distinct systems and networks, and how to validate them in simplified “artificial” functional assays and models. Functional follow-up studies of the discovered networks and molecules in the right context are required to obtain specific functional annotations as discussed in *4.6*. A potential approach to gain full mechanistic insight will require coordinated sets of molecular and cellular multilayer omics data obtained at multiple time points and collected from disease-relevant tissues representing different stages of damage or repair. Additionally, combination of different omics in different human compartments, and combination of omics in the human disease with animal models may help to assess the biological significance (55, 81). However, such combination of omics techniques needs high-level integration. Combination of data-driven and knowledge-driven models into integrative models may define, whether the altered pathways are related to cause or effect. Here, *in situ* RNAseq will also help in elucidating these aspects of cell-cell interaction without the need of artificial *in silico* and *in vitro* modeling.

**Figure 9:**
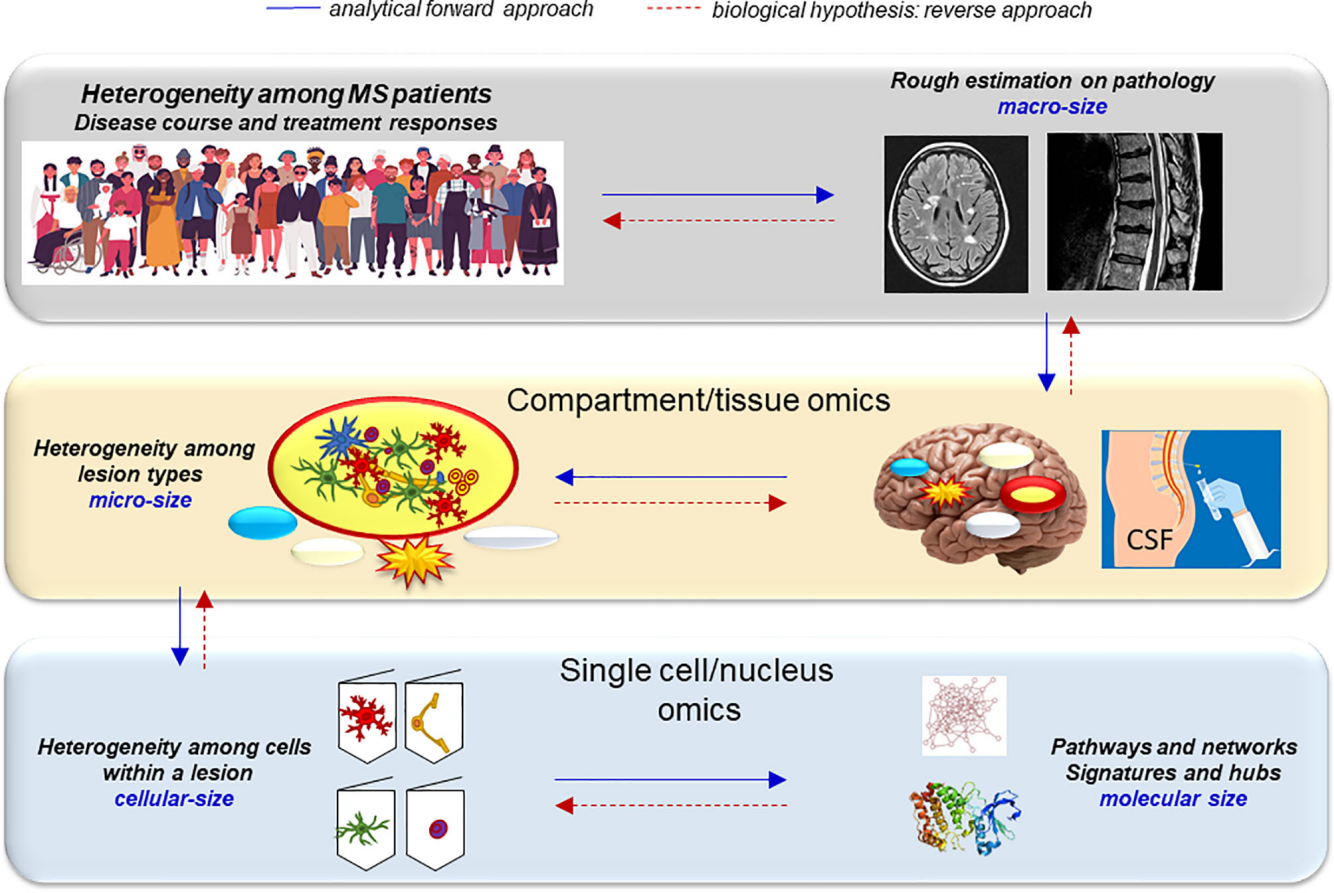
Decoding the heterogeneity of MS with a reverse genetics approach. Analytical forward approach (blue arrows): The heterogeneity of the MS population is reflected by the heterogeneous course of MS and treatment responses. The hallmark of MS, WM brain lesions look similar on conventional MRI scans, but their histopathology is very different: characterized as active, inactive, chronic active and remyelinating/repairing lesions. This heterogeneity is most likely caused by the different cell types present in the lesions that is controlled by the heterogeneity of different networks and pathways activated within the cells and determined by some major hubs and molecular signatures. Biological hypothesis, reverse approach (red arrows): To decode this complexity, a reversed biological approach can be an alternative strategy. It can start from genetic regulation and molecular changes within individual cells that contribute to their fate. This will determine the evolution of lesions, and such complexity of lesion types will determine the individual MS brain and clinical outcomes. MS fate thus ultimately may depend on the interaction of singular cells.

With the rapid acquirement of data, the concept to understand the heterogeneity of MS may change, starting from the causative molecular signature rather than the clinical phenotype (90). The classic approach (*analytical forward approach* **)** (**Figure 9**) applies omics of a patient group with a particular phenotype and determines, which variants these people have in common. In contrast, analyses may also start from large omics datasets by examination what human variants have in common in a clinical setting and connect it to endophenotypes (*biological reverse approach*) (**Figure 9**). Applying this latter strategy for understanding the mechanism behind MS phenotypes, the interaction of functional subsets of single cells and their unique intracellular systems should be analyzed, where macromolecules and key hubs interact with each other in networks. The observed heterogeneity of cell subtypes (endophenotypes) in individual MS lesions may be responsible for the evolution of different lesion types, and the heterogenous composition of these lesion types may contribute to changes in specific brain networks that are ultimately responsible for the clinical heterogeneity (**Figure 9**). However, here the snapshot problem will also still be an obstacle.

#### 4.7.4 Clinical/Pathological Classification *vs* Tissue Systems Biology

Finally, MS disease classification is only based on clinical phenotypes and not endophenotypes. Differential signatures in the CSF may reflect the presence of particular lesion types in the brain but also highlight the heterogeneity of lesion/pathogenesis subtypes (endophenotypes) in phenotypically similar patient groups. However, such heterogeneity may also arise from the timepoint of sampling. Avoiding this, repeated analyses of samples with large sample size are needed. While solving the “snap-shot problem”, and also adding the endophenotypic signatures for the patients may specify the pathological events, and thereby use more targeted therapies. A recent study found strong association between severe cortical pathology and a distinctive CSF inflammatory profile (91). Additionally, using positron-emission tomography (PET), potential future targets for biomarkers could be identified in different MS lesion types *in vivo*. By combining different sources of information, such as omics and structural/functional neuroimaging, it may be possible to obtain a new integrated picture of the pathophysiological process in MS that could span from molecular alterations to cognitive manifestations.

## 5 Conclusion

Systems biology approach on MS brain tissue may not yet have reached as far as hoped due to tissue availability including different tissue sampling, divergent methodologies, analytic obstacles, gaps of data, and integration of datasets from various sources. Therefore, despite omics studies in MS have been present for decades, it can still be difficult to present an economical summary. However, it clearly revealed that MS is a global brain disease, where all resident brain cells are altered in different degrees. It showed that MS is a more complex and heterogenous disease on molecular level compared to the clinical classification. Paradoxically, this is also reflected in the difficulties of finding validated biomarkers based on omics approaches. Defining endophenotypes may help to disentangle the observed heterogeneity and find common patterns and dysregulated pathways: overcoming the snapshot problem is necessary for such functional interpretations.

Some of the consistent and/or key findings achieved by the systems biology investigations are inflammation within the brain of progressive MS, high levels and multiple types of HLA expression, high neuronal changes in both WM and GM, where TNF signaling is important and that CUX-2-expressing neurons are the most vulnerable; marked oligodendrocyte heterogeneity in the different WM lesion types; pathological/molecular changes in microglia within the NAWM before lesion evolution and distinct functional subgroups during lesion evolution; different astrocyte and microglia polarizations even in slowly expanding lesion rim; and high expression of CXCL12, SCD, STAT6, CD163 and TGFβR2 in all types of WM lesions.

The main power of systems biology is the comprehensive and unbiased approach at a time when out-of-the-box hypotheses for the disease course and progression are needed. Omics-driven data in MS are exponentially growing and if solutions to the major limitations (e.g. sample size, snap-shot problem) are solved, novel hypothesis-driven data can emerge. Applying innovative integrative methods to tissue and single-cell multi-omics combined with extensive interdisciplinary and international collaboration is a logical step forward. This will help give direction for functional experiments and in-depth molecular biological studies.

## Data Availability Statement

The original contributions presented in the study are included in the article. Further inquiries can be directed to the corresponding author.

## Author Contributions

ME made the systematic article search and wrote the manuscript and made the figures. ZI provided critical feedback and helped shape the manuscript. JB and RR came with input and comments to the manuscript. All authors contributed to the article and approved the submitted version.

## Conflict of Interest

The authors declare that the research was conducted in the absence of any commercial or financial relationships that could be construed as a potential conflict of interest.

## Publisher’s Note

All claims expressed in this article are solely those of the authors and do not necessarily represent those of their affiliated organizations, or those of the publisher, the editors and the reviewers. Any product that may be evaluated in this article, or claim that may be made by its manufacturer, is not guaranteed or endorsed by the publisher.
